# Assessment of the Effects of Bisphenol A on Dopamine Synthesis and Blood Vessels in the Goldfish Brain

**DOI:** 10.3390/ijms20246206

**Published:** 2019-12-09

**Authors:** Qing Wang, Fangmei Lin, Qi He, Xiaochun Liu, Shiqiang Xiao, Leyun Zheng, Huirong Yang, Huihong Zhao

**Affiliations:** 1 College of Marine Sciences, South China Agricultural University, Guangzhou 510642, China; wangqing@scau.edu.cn (Q.W.); linmu212422@163.com (F.L.); 13657405840@163.com (Q.H.); zhuifeng2019yun@163.com (X.L.); xiaosq01@haid.com.cn (S.X.); 2 Joint Laboratory of Guangdong Province and Hong Kong Region on Marine Bioresource Conservation and Exploitation, College of Marine Sciences, South China Agricultural University, Guangzhou 510642, China; 3 Fisheries Research Institute of Fujian, Xiamen 361000, China; heicaonan@163.com

**Keywords:** bisphenol A, transcriptome, dopamine, signaling pathway, brain blood vessels

## Abstract

Bisphenol A (BPA) is an abundant contaminant found in aquatic environments. While a large number of toxicological studies have investigated the effects of BPA, the potential effects of BPA exposure on fish brain have rarely been studied. To understand how BPA impacts goldfish brains, we performed a transcriptome analysis of goldfish brains that had been exposed to 50 μg L^−1^ and 0 μg L^−1^ BPA for 30 days. In the analysis of unigene expression profiles, 327 unigenes were found to be upregulated and 153 unigenes were found to be downregulated in the BPA exposure group compared to the control group. Dopaminergic signaling pathway-related genes were significantly downregulated in the BPA exposure group. Furthermore, we found that serum dopamine concentrations decreased and TUNEL (terminal deoxynucleotidyl transferase 2-deoxyuridine, 5-triphosphate nick end labeling) staining was present in dopamine neurons enriched regions in the brain after BPA exposure, suggesting that BPA may disrupt dopaminergic processes. A KEGG analysis revealed that genes involved in the fluid shear stress and atherosclerosis pathway were highly significantly enriched. In addition, the qRT-PCR results for fluid shear stress and atherosclerosis pathway-related genes and the vascular histology of the brain showed that BPA exposure could damage blood vessels and induce brain atherosclerosis. The results of this work provide insights into the biological effects of BPA on dopamine synthesis and blood vessels in goldfish brain and could lay a foundation for future BPA neurotoxicity studies.

## 1. Introduction

Bisphenol A (BPA) is an environmental contaminant that is widely used in many consumer goods. For example, it is used in coatings on food cans, thermal receipt paper, and water bottles [1,2]. It is estimated that the production of the biosurfactant market could increase to 460 million kilograms by 2020, indicating that approximately 450,000 kg of BPA will be released into the environment [3]. BPA levels are very high in water worldwide. For example, levels of BPA in surface water in the Netherlands reached 21 μg L^−1^ in the summer [4]. The level in landfill leachate from Japan was as high as 17.2 mg L^−1^ [5]. Owing to its release from used polycarbonate animal cages, the concentration of BPA increased to 310 μg L^−1^ [6]. In China, BPA levels in water are also up to 370 μg L^−1^ [7,8]. The aquatic environment contains large amounts of BPA, and this chemical can enter aquaculture water, mainly through the natural degradation of BPA-containing products and landfill leachate and sewage treatment wastewater contamination [9,10,11,12,13,14].

Fish are often used to evaluate the health of aquatic ecosystems, in particular, physiological and biochemical changes in fish are considered biomarkers of environmental pollution [15]. Previous studies have suggested that BPA, as an analog of estrogen, may exert an estrogenic effect on fish gonad development and reproductive axis-related gene expression [16,17,18,19,20]. BPA also affects various biological processes in fish, including metabolism, individual cortisol stress responses, energy metabolism, muscle structure, motor behavior, and axonal growth, thereby affecting fish growth and development [21,22,23,24]. In mammals, studies have shown that BPA is neurotoxic and induces brain, region-specific changes in dopaminergic processes, resulting in hyperactivity and attention deficits [25,26]. While numerous toxicological studies have described the effects of BPA on fish, few have investigated the effects of BPA on fish brain.

Our previous study showed that 50 μg L^−1^ BPA exposures exert an obvious physiological effect on goldfish, such as disrupted testis maturation and reduced ovarian maturation, by affecting the HPG (Hypothalamic-pituitary-gonadal) axis [19]. This concentration can also be detected in aquatic environments [7,8]. Thus, in the present study, we perform a deep transcriptome analysis of the brains of goldfish exposed to 50 μg L^−1^ BPA to investigate the effects of BPA on fish brain. The data reveal candidate pathways for genes associated with dopamine synthesis, brain blood shear stress, and atherosclerosis, and provide extensive information about novel gene sequences and gene expression profiles. Additionally, we analyze serum dopamine concentrations and the expression profiles of the dopaminergic signaling pathway-related genes tyrosine hydroxylase (*th*), monoamine oxidase (*mao*), dopamine 1 receptor (*dr1*), dopamine 2 receptor (*dr2*), and dopamine transporter (*dat*). We also examine apoptosis in dopaminergic neurons with a terminal deoxynucleotidyl transferase 2′-deoxyuridine 5′-triphosphate nick end-labeling (TUNEL) assay. The results show that BPA can disrupt dopamine synthesis. Additionally, the expression profiles of blood shear stress and atherosclerosis genes, and the results of brain vascular histologic analysis, show that BPA may damage brain blood vessels. The results of this study will help guide further investigation into the dopamine system and brain blood vessels in goldfish.

## 2. Results

### 2.1. Sequencing and Assembly

All goldfish in the treatment group were treated with BPA for 30 days prior to any testing. Six cDNA libraries were constructed: Three control group and three treatment group libraries. A total of 126,926,073 raw reads were generated. There were 62,210,962 and 63,902,684 clean reads in the control and treatment group libraries, respectively. The Q20 values were greater than 98%, and the GC (GC base) content was 49% (Table S1). The low-quality reads were removed, and the clean reads were used for sequence assembly. After processing with Trinity software, 533,674 unigenes were ultimately generated (Table S2). These results suggested that high-quality transcriptome data were obtained. The sequence data for the unigenes were deposited in the Sequence Read Archive (SRA) of NCBI under accession numbers SRR8439278 to SRR8439283.

### 2.2. Functional Analysis

To evaluate the functions of the unigenes, 380,155 unigene sequences were aligned with sequences in seven public databases. The number of successfully annotated unigenes was 25,738 for the Nr database, 74,201 for the KEGG database, 96,930 for the Swiss-Prot database, and 13,933 for the GO database (Table S3).

### 2.3. Analysis of Differential Gene Expression

**Figure 1:**
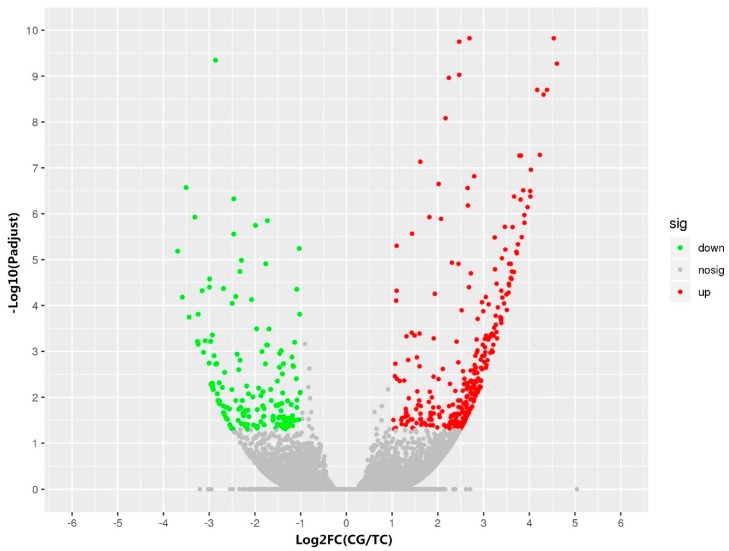
Volcano plot of the DEGs (differentially expressed genes) between the control group (CG) and the Bisphenol A (BPA) treatment group (TG). Log2FC (CG/TG) indicates the mean expression level (log2 fold change) for each gene. Each dot represents a gene. The red dots represent upregulated genes, green dots represent downregulated genes, and gray dots represent genes that were not differentially expressed.

DESeq2 software was used to analyze the unigene data and to identify the genes with significant expression differences between the control group and the treatment group. Genes with expression fold changes >2 (*p* < 0.05) were considered to be differentially expressed. A total of 481 genes were differentially expressed, including 327 upregulated genes and 153 downregulated genes (Figure 1). Among these DEGs (differentially expressed genes), the expressions of three dopaminergic signaling pathway-related genes, *th*, *mao,* and *dr1*, were significantly lower in the BPA-exposed group than in the control group (Figure 3A). The details of the changes in DEGs between the groups are listed in Table S4.

### 2.4. GO and KEGG Enrichment Analysis

To further analyze the DEGs between the control group and the BPA treatment group, the DEGs were annotated with the GO and KEGG databases. A total of 308 DEGs between the two groups were annotated with one or more GO terms (Figure S1). In the GO enrichment analysis, the most represented term in the biological processes category was the cellular process (GO: 0009987). In the cellular component category, the most strongly represented term was the extracellular region part (GO:0044421) term. In the molecular function category, nucleic acid binding transcription factor activity (GO:0001071) was the major term. A number of unigenes were also involved in metabolic processes, developmental processes, rhythmic processes, cell junctions, and catalytic activity, suggesting that these unigenes may play roles in mediating the effects associated with BPA exposure.

**Figure 2:**
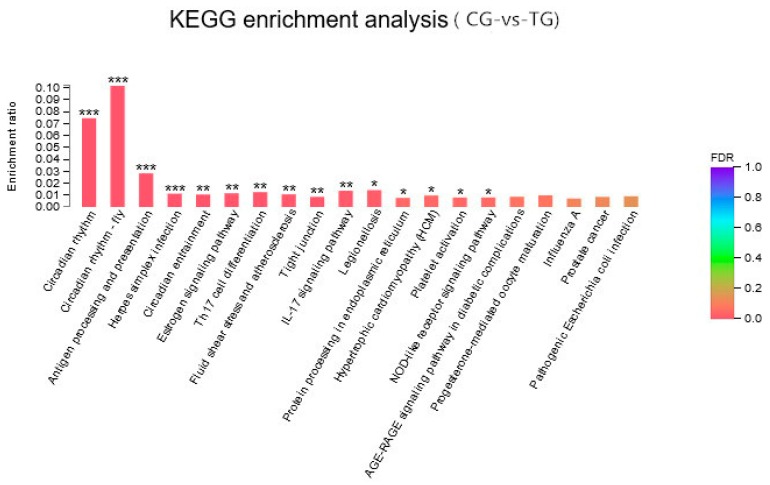
Enriched KEGG terms for the DEGs between the control group (CG) and the BPA treatment group (TG). * indicates a significant difference at *p* < 0.05, ** indicates a significant difference at *p* < 0.01, and *** indicates a significant difference at *p* < 0.001.

In KEGG analyses, for any pairwise comparisons, the most highly enriched KEGG pathways were associated with circadian and immune processes; these pathways included the circadian rhythm, antigen processing and presentation, and herpes simplex infection pathways. In addition to these pathways, the fluid shear stress and atherosclerosis pathway was also highly significantly enriched (Figure 2).

Among the DEGs in the fluid shear stress and atherosclerosis pathway, *hsp90* and *calm*, which are associated with endothelial cell (EC) caveolae and vascular smooth muscle contraction, were downregulated in the BPA exposure group. The *β-catenin* and *mek5* genes, which are associated with antiatherogenic processes, were also downregulated, as was *F-actin*, which is associated with cytoskeletal alignment. *Et-1*, which is associated with proatherogenic processes, was upregulated in the BPA exposure group (Figure 4A, Table S5, Figure S2).

### 2.5. Expression Profiles of Genes in Dopaminergic Signaling Pathway and the Fluid Shear Stress and Atherosclerosis Pathway

**Figure 3:**
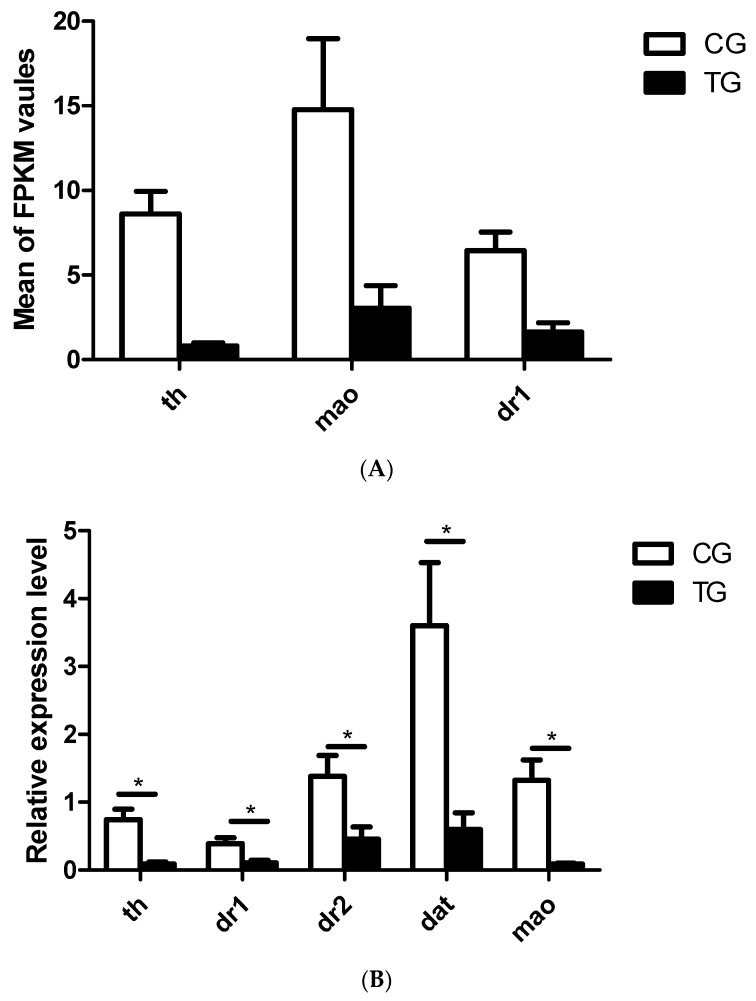
DEGs in the dopaminergic signaling pathway and analysis of the expression of dopaminergic signaling pathway-related genes by qRT-PCR. (**A**) Expression levels of 3 DEGs in the dopaminergic signaling pathway from the transcriptome data. (**B**) Expression levels of 6 dopaminergic signaling pathway-related genes determined by qRT-PCR (*n* = 3). CG: Control group; TG: BPA treatment group; and FPKM: Fragments per kilobase of transcript per million mapped reads. The qRT-PCR results were calculated from at least three independent experiments. EF1 was used as an internal normalization control. The data are expressed as the mean ± SD (* *p* < 0.05).

**Figure 4:**
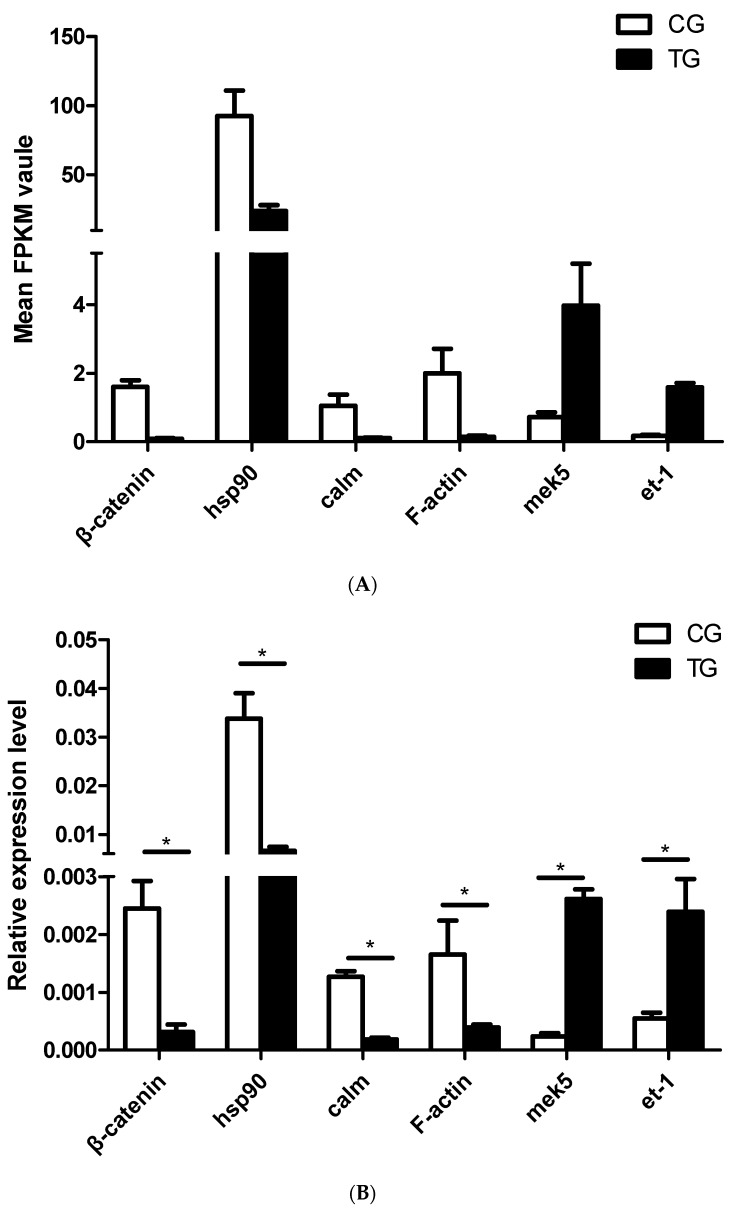
DEGs in the fluid shear stress and atherosclerosis pathway and validation by qRT-PCR. (**A**) Expression levels of 6 DEGs in the fluid shear stress and atherosclerosis pathway from the transcriptome data. (**B**) Validation of DEGs in the fluid shear stress and atherosclerosis pathway by qRT-PCR (*n* = 3). CG: Control group; TG: BPA treatment group; and FPKM: Fragments per kilobase of transcript per million mapped reads. The qRT-PCR results were calculated from at least three independent experiments. EF1 was used as an internal normalization control. The data are expressed as the mean ± SD (* *p* < 0.05).

As described in the Introduction Section, the effects of BPA on dopaminergic pathways in mammals have been described in previous studies [25,26]. In the current study, we therefore focused on the BPA effects on dopaminergic pathways in fish. The expression levels of key genes in the dopaminergic signaling pathway and the fluid shear stress and atherosclerosis pathway, including *th*, *dr1*, *dr2*, *dat*, *mao*, *β-catenin*, *hsp90*, *calm*, *mek5*, *F-actin,* and *et-1*, were analyzed. The results showed that the expression of all of the dopaminergic signaling pathway-related genes, including *th*, *dr1*, *dr2*, *dat*, and *mao*, was significantly lower in the BPA exposure group than in the control group (Figure 3B). The fluid shear stress and atherosclerosis pathway-related genes, such as *β-catenin*, *hsp90*, *calm,* and *F-actin*, were significantly downregulated in the BPA exposure group compared to the control group, but the expression of *mek5* and *et-1* was increased after BPA exposure (Figure 4B).

### 2.6. Analysis of Dopamine Concentrations and Brain Dopaminergic Neuron Apoptosis

**Figure 5:**
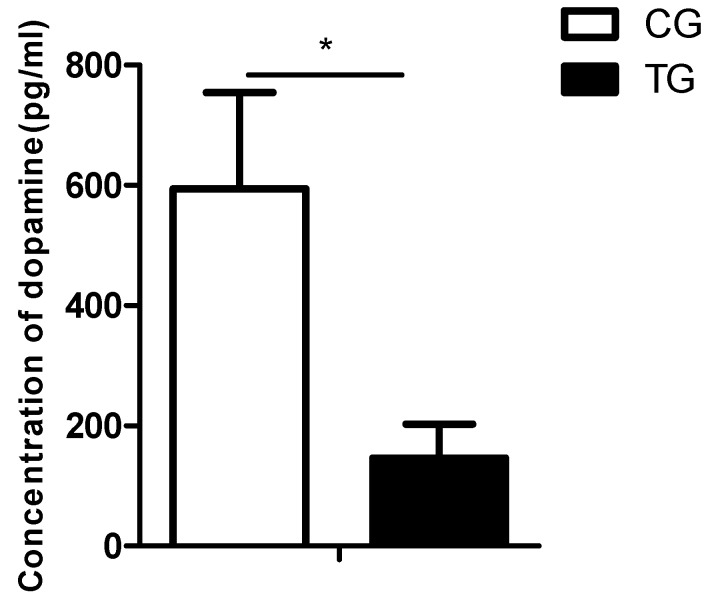
Serum dopamine concentrations in the control group (CG) and the BPA treatment group (TG). The data are expressed as the mean ± SD (*n* = 3); * indicates a significant difference at *p* < 0.05.

BPA has previously been shown to affect dopaminergic pathways in mammals [25,26], and we showed here that DEGs involved in dopaminergic pathways were deregulated in goldfish after BPA exposure. To determine whether BPA affected dopaminergic production and dopaminergic neurons in goldfish in vivo, we examined the effects of BPA exposure on serum dopamine concentrations and stained brain sections with TUNEL. Serum dopamine concentrations were examined. Compared with those in the control group, dopamine concentrations were significantly decreased in the BPA-exposed group (Figure 5).

**Figure 6:**
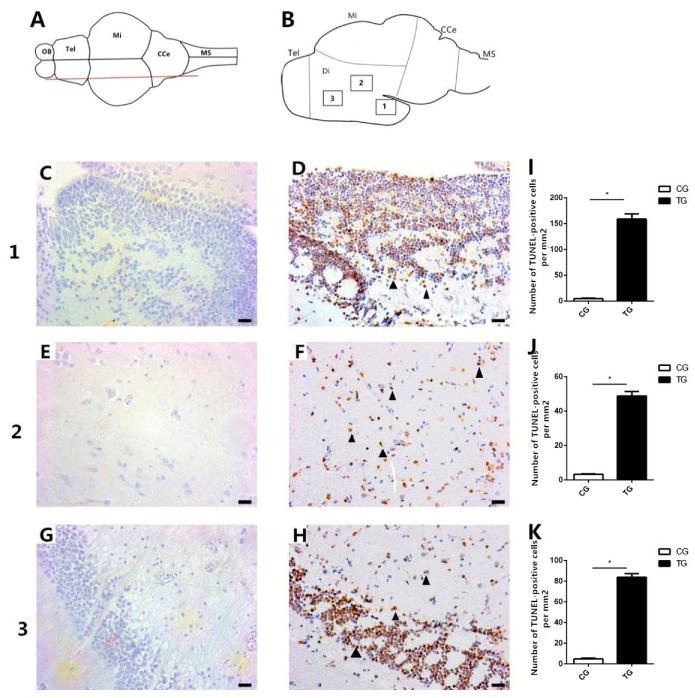
TUNEL staining of the brains of goldfish in the control group (0 μg L^−1^) and the BPA treatment group (50 μg L^−1^). (**A**) Dorsal view of the fish brain; the red line represents the slice position. (**B**) Longitudinal sectional view of the fish brain; the boxed areas represent the positions of the sections. (**C**–**H**) TUNEL staining of brain Section 1, Section 2, and Section 3. No TUNEL-positive signals were detected in the control group in the three positions (**C**,**E**,**G**). (**D**,**F**,**H**) TUNEL-positive signals were detected in all three positions in the BPA-exposed group (black arrowheads). (**I**–**K**) Statistical analysis of the number of TUNEL-positive cells per mm^2^. Five images per fish were analyzed. * indicates a significant difference (*n* = 3). Tel: Telencephalon; OB: Olfactory bulb; Di: Diencephalon; Mi: Midbrain; CCe: Corpus cerebelli; and MS: Medulla spinalis. Scale bars, 50 μm.

In the treatment group, positive TUNEL staining was found in the diencephalon area, the main site of dopaminergic neuron distribution (Figure 6D,F,H), and the number of TUNEL-positive cells per unit area was also higher in the BPA treatment group (Figure 6I–K), but no positive signals were observed in the corresponding area in the control group (Figure 6C,E,G). The main dopaminergic neuron distribution area has been described by Matsui [26] and Schweitzer [27]. The dorsal view of the fish brain is shown in Figure 6A, and the longitudinal sectional view is shown in Figure 6B.

### 2.7. Vascular Histology of the Brain

**Figure 7:**
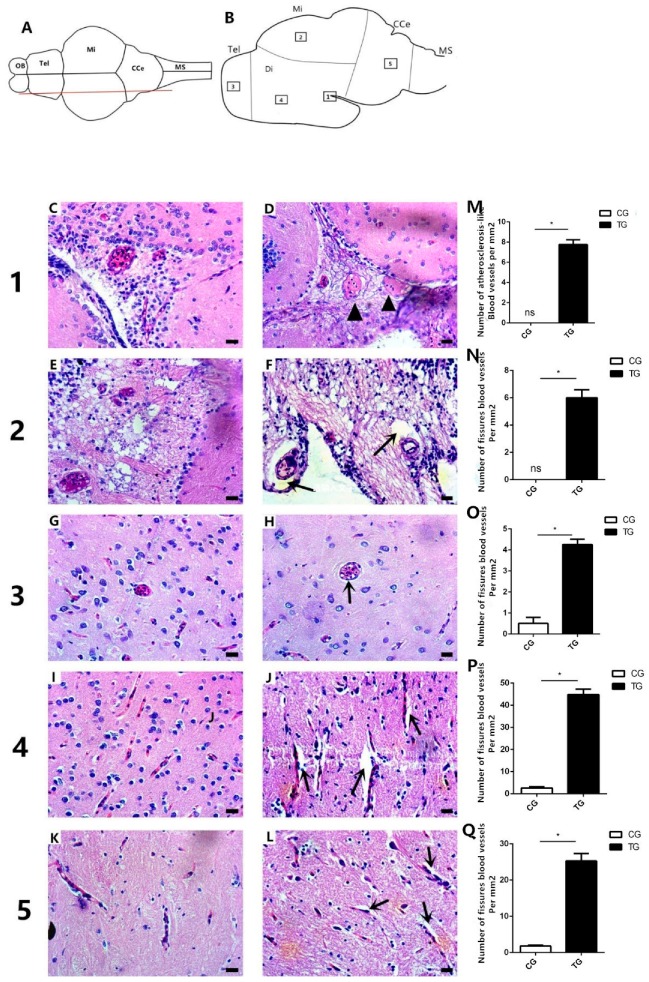
Histology of the brains of goldfish from the control group (0 μg L^−1^) and the BPA treatment group (50 μg L^−1^). (**A**) Dorsal view of the fish brain; the red line represents the slice position. (**B**) Longitudinal sectional views of the fish brain; the boxed areas represent the positions of the sections. (**C**–**L**) Histology of the brain in the five areas. (**C**,**E**,**G**,**I**,**K**) Brain histology of the five areas in the control group. (**D**,**F**,**G**,**J**,**L**) Brain histology of the five areas in the BPA-exposed group. Atherosclerosis was found in the diencephalon (black arrowheads) in the BPA-exposed group (**D**). Fissures (black arrows) were found in the midbrain (**F**) and telencephalon (**G**) region, as well as in capillaries in the telencephalon (**J**) and corpus cerebelli (**L**). (**M**) Statistical analysis of the number of fissured blood vessels per unit area. * indicates a significant difference (*n* = 3). (**N**–**Q**) Statistical analysis of the number of atherosclerosis-like vessels per mm^2^. Five images per fish were analyzed. * indicates a significant difference (*n* = 3). Tel: Telencephalon; OB: Olfactory bulb; Di: Diencephalon; Mi: Midbrain; CCe: Corpus cerebelli; and MS: Medulla spinalis. Scale bars, 50 μm.

To determine whether BPA affects blood vessels and induces atherosclerosis in the brain, brain histological analysis was conducted. In the diencephalon area, the blood vessels in the control group had large vascular lumens and were filled with red blood cells, but the blood vessels in the BPA exposure group showed vascular occlusion and had only few red blood cells (Figure 7C,D); the images of the H&E staining of atherosclerotic pathology was referred to in Paralichthys albigutta (Figure S3). In the midbrain, there were large fissures between blood vessels and the surrounding tissue in the treatment group (Figure 7F,N), but no fissures were observed in the control group (Figure 7E,N). Fissures were also observed around blood vessels in the telencephalon region and around capillaries in the diencephalon and corpus cerebellum regions after BPA exposure (Figure 7H,J,L); statistical analysis showed that the number of fissures blood vessels per unit area was also higher in the BPA treatment group (Figure 7N–Q), but no fissures were observed in these areas in the control group brains (Figure 7G,I,K). Additionally, we examined the vessel wall thickness in similar anatomical areas and found that the blood vessel walls were thicker in the BPA-treated group than in the control group (Table 1). These results suggested that BPA exposure might damage brain blood vessels and induce atherosclerosis.

## 3. Discussion

BPA is widely found in aquatic environments and causes reproductive toxicity in fish, often resulting in abnormal testicular and ovarian development [16,17,18,28,29]. BPA has also been demonstrated to accumulate in various fish tissues, leading to pathological changes, including changes in the endocannabinoid system, changes in the ontogeny of the cortisol stress response, and energetic impairments [22,23,30].

Several reports have shown the neurotoxic effects of BPA in a variety of animal models [20,31,32,33,34,35,36,37]. However, the toxic effects of BPA on fish brain development and related gene expression have rarely been studied. In the present study, we found that BPA disrupts dopaminergic processes, including increasing the apoptosis of dopamine neurons enriched brain regions, decreasing serum dopamine concentrations, and downregulating genes involved in dopaminergic signaling pathways. Studies have indicated that dopamine neurons are vulnerable to oxidative damage, and many studies have shown that BPA is an exogenous estrogen with oxidative toxicity and it can cause tissue oxidative damage [38,39,40,41,42,43,44]. Thus, we propose that BPA may affect dopamine synthesis via oxidative damage to dopamine neurons. Furthermore, we detected the increase of apoptosis in dopamine neurons-enriched brain regions by TUNEL, and the results show that BPAs have oxidative damage and may cause the apoptosis of dopamine neurons. In addition, the dopamine system is complex, and its normal function involves many components, such as dopamine metabolic enzymes (TH, MAO), the dopamine transporter (DAT), dopamine receptor (DR), and three signaling pathways of the dopamine system. Changes in any of these factors affect dopamine synthesis. Our results showed that BPA exposure significantly decreased the serum dopamine concentrations and the expressions of *th, mao, dat, dr1,* and *dr2*. These changes may explain the effects of BPA that induced the damage (TUNEL) to the dopamine neurons and further induced the decrease of dopamine levels and the expression of genes involved in dopamine synthesis. There is a possibility that some TUNEL-positive cells are not dopaminergic cells, because the loss of D1 receptors is also found in addition to DAT and D2. Thus, further research is needed.

In the present study, the KEGG pathway enrichment analysis was shown to be toxic to blood vessels in the brains of BPA-exposed fish. After BPA exposure, the expression of genes related to stable blood flow with laminar shear stress (*hsp90*, *calm*, *β-catenin,* and *F-actin*) was decreased, while the expressions of *mek5* and *et-1*, genes related to disturbed blood flow with low/oscillatory shear stress, was increased, suggesting that BPA exposure weakened the stabilization of blood flow and strengthened the disruption of blood flow. Furthermore, several DEGs relating to the immune function were altered, especially in pathways relating to herpes simplex infections. These results suggest that BPA may affect immune functions in fish; however, further research is needed to establish this.

The shape of blood vessels is strongly influenced by blood shear stress, with shape changes preferentially occurring in areas of disturbed flow or low shear stress [45,46,47,48]. The shear stress of blood in the brain is affected by vascular ECs (Endothelial Cells); these ECs, which cover the inner surface of blood vessels, are constantly exposed to shear stress because of the frictional force created by blood flow. Previous studies have shown that altered gene expression in ECs changes EC caveolae, inducing changes in local shear stress [49,50]. In addition, shear stress can alter smooth muscle cells and affect vascular smooth muscle contraction [51,52], thus weakening vascular smooth muscle contraction and causing blood vessel deformation (such as the fissures observed in the brain histological analysis) (Figure 7). In addition, a previous study has shown that increases in fluid shear stress increased *mek5* expression in ECs [53]. Our study showed that, after BPA exposure, *mek5* expression was increased, indicating that the vascular pressure in goldfish brain was increased. The vascular histology showed that the brain blood vessels had atherosclerosis in BPA-exposed fish. We propose that BPA exposure may disrupt gene expression in vascular ECs and thus weaken vascular smooth muscle contraction, destroying the shape of blood vessels and resulting in atherosclerosis, but more research may be needed.

## 4. Materials and Methods

### 4.1. Animals and Treatments

Juvenile goldfish (mean body length, 6.5 ± 0.69 cm; mean body weight, 9.4 ± 1.23 g) were obtained from a fish farm in Guangzhou, China, and were maintained in aquaria. The concentration of BPA used in this study was 50 μg L^−1^. The exposure experiments were carried out in six different glass tanks; 3 tanks were used for each exposure group, with 30 fish per tank. The treated group was exposed to 50 μg L^−1^ BPA for 30 days. After the exposure, 15 fish were randomly selected from each group, anesthetized with MS-222 (Sigma-Aldrich, Saint Louis, MO, USA) and then sacrificed via decapitation. The brains of three fish in each group were collected, and the total RNA was extracted from each brain. The RNA from the brains of three fish from each tank was mixed into one pooled RNA sample for a total of three pooled samples per group. Then, the six pooled RNA samples (three control group samples and three treatment group samples) were used to construct a transcriptome library. The brains of six of the fifteen fish sacrificed from each group were dissected and fixed for histological analysis. The brains of the other 6 fish were collected, flash-frozen in liquid nitrogen, and then stored at −80 °C. At the same time, blood samples were collected, and pairs of samples were mixed to form pooled serum samples after centrifugation. All animal experiments were conducted in accordance with the guidelines and approval was obtained from the appropriate Animal Research and Ethics Committees of South China Agricultural University (SHAUQ201810, 15 April 2018).

### 4.2. RNA Extraction and Library Preparation

The brain samples were lysed in TRIzol reagent (Invitrogen, Carlsbad, CA, USA) for RNA extraction according to the manufacturer’s instructions. Agarose gels (1.5%) were used to examine RNA degradation and contamination. RNA concentrations and integrity were examined with a Qubit RNA Assay Kit in a Qubit 2.0 Fluorometer (Life Technologies, Carlsbad, CA, USA) and with an RNA Nano 6000 Assay Kit in an Agilent Bioanalyzer 2100 System (Agilent Technologies, Santa Clara, CA, USA).

RNA (2 µg per sample) was used as input material for RNA sample preparation. In short, mRNA was isolated from total RNA using magnetic beads with bound poly(T) oligonucleotides, and the purified mRNA was then cut into small fragments at high temperatures using bivalent cations.

First-strand cDNA was synthesized with a random hexamer primer using the M-MuLV Reverse Transcriptase kit (MBI, Fermentas, Lithuania); Second-strand cDNA was then synthesized for the first-strand fragments. Double-stranded cDNA was synthesized using a SuperScript double-stranded cDNA synthesis kit (Invitrogen, Carlsbad, CA, USA) with random hexamer primers (Illumina). Then, the synthesized cDNA was subjected to end-repair, phosphorylation and ‘A’ base addition according to Illumina’s library construction protocol. cDNA fragments of 150–200 bp were preferentially selected, and PCR amplification was performed for 15 cycles. Finally, the PCR products were purified and evaluated using an Illumina HiSeq 2500 platform.

### 4.3. Transcriptome Assembly and Gene Functional Annotation

Raw reads were obtained after the removal of reads containing the adaptor sequence, reads containing poly-N sequences and low-quality reads with SQ values <20 and nucleotide ratios greater than 50%. Only sequences with Phred quality scores >20 were selected for downstream analysis. The selected reads were assembled into transcripts using Trinity software [54]. Then, TGICL V2.1 software [55] was used to process the contigs; redundant sequences were removed and additional assembly was performed.

Unigenes were ‘blasted’ (searched) against the Nr database, the Kyoto Encyclopedia of Genes and Genomes (KEGG) database, the Swiss-Prot database, and the EuKaryotic Orthologous Groups (KOG)/Clusters of Orthologous Groups (COG) database using BLASTx. In addition, the protein sequences were obtained, and the functional annotation information was obtained according to the protein sequences.

### 4.4. Differential Expression Analysis

Differential expression analysis was identified using DESeq2 version 1.4.5 [56]. The Benjamini and Hochberg method was used to adjust the obtained *p*-values [57]. Genes with fold changes >2 and *p* < 0.05 were considered differentially expressed.

### 4.5. Gene Ontology (GO) and KEGG Enrichment Analyses

GOseq V1.16.2 software was used to perform GO enrichment analysis of differentially expressed genes (DEGs) [58]. Then, KOBAS software was used to test the statistical enrichment of the DEGs in the KEGG pathways [59].

### 4.6. RNA Isolation, Reverse Transcription and qRT-PCR

Total RNA was extracted using TRIzol reagent and then reverse transcribed using a Transcriptor First Strand cDNA Synthesis Kit (Toyobo, Tsuruga, Japan) according to the manufacturer’s instructions. SYBR Green I Master Mix (Roche, Basel, Switzerland) was used to conduct qRT-PCR analysis on a Roche LightCycler 480 Real-Time PCR System. The PCR conditions were as follows: 95 °C for 5 min for activation followed by 40 cycles of 95 °C for 20 s, 58 °C for 20 s, and 72 °C for 20 s. The *ef1* gene was used as the internal control. The expression of each target gene was normalized by the 2^−∆∆*C*t^ method [60]. The primers are listed in Table S6.

### 4.7. Dopamine Detection

Serum dopamine was measured using a Fish Dopamine ELISA Kit (Cusabio, Wuhan, China), following the manufacturer’s instructions. A Synergy H4 Hybrid Multi-Mode Microplate Reader (BioTek, Winusky, USA) was used to detect the optical density per well at 450 nm within 10 min.

### 4.8. Brain Histology

Brains were dissected from fish in the control group and treatment group, fixed in Bouin’s fluid for 24 h at room temperature, dehydrated, and embedded in paraffin wax. Subsequently, the samples were subjected to histological analysis through hematoxylin and eosin (H & E) staining.

### 4.9. TUNEL Staining

TUNEL analysis was performed, as described in a previous study [61]. Brain apoptosis was detected with a TUNEL assay kit (Roche, Basel, Switzerland) according to the manufacturer’s instructions. After TUNEL staining, hematoxylin was used to restain the tissues. Photographs were obtained under a Nikon optical microscope (Nikon, Sendai, Japan).

## 5. Conclusions

In this study, transcriptome analysis of the brains of goldfish exposed to BPA was performed using RNA-Seq. Upon comparison of unigene expression profiles, 327 unigenes were found to be upregulated and 153 unigenes were found to be downregulated in the BPA exposure group compared to the control group. Among these DEGs, dopaminergic signaling pathway-related genes were significantly downregulated in the BPA-exposed group compared to the control group. Furthermore, TUNEL analysis revealed that BPA exposure induced dopamine neuron enriched regions in brain apoptosis, and dopamine concentrations also decreased after BPA exposure, indicating that BPA can disrupt dopaminergic processes. KEGG analysis showed that the DEGs were highly enriched in the fluid shear stress and atherosclerosis pathway. In addition, qRT-PCR analysis of DEGs in the fluid shear stress and atherosclerosis pathway and analysis of the vascular histology of the brain showed that BPA exposure can damage brain blood vessels. We could greatly speculate that BPA exposure globally affects the DA system, particularly in the diencephalon, which is known as the most DA enriched region, and leads to the reduction of serum DA levels in goldfish.

## Figures and Tables

**Table 1:** Summary of vessel wall thicknesses in similar anatomical areas for the control group (CG) and treatment group (TG).

Sample	Areas				
	1	2	3	4	5
CG	3.52 ± 0.07 µm *	3.73 ± 0.07 µm *	0.89 ± 0.03 µm	0.81 ± 0.03 µm	0.92 ± 0.02 µm *
TG	2.31 ± 0.04 µm	2.54 ± 0.05 µm	0.71 ± 0.02 µm	0.62 ± 0.01 µm	0.67 ± 0.02 µm

The results are expressed as the mean ± SD (*n* = 3). Significant differences between CG and TG are identified with *. Numbers 1, 2, 3, 4, and 5 correspond to the brain anatomical areas shown in Figure 7.

## Supplementary Materials

Supplementary materials can be found at https://www.mdpi.com/1422-0067/20/24/6206/s1.
