# Supplementary material for: Assessment of the Effects of Bisphenol A on Dopamine Synthesis and Blood Vessels in the Goldfish Brain
Source: Int J Mol Sci. 2019 Dec 9;20(24):6206. doi: 10.3390/ijms20246206 (PMC6941070; doi:10.3390/ijms20246206)
Supplement: Supplementary file 1 [file ijms-20-06206-s001.pdf]

International Journal of
Molecular Sciences

MDPI

Table S1. Summary of RNA-seq data for control group (CG) and treatment group (TG).

Sample	Raw reads	Clean reads	Q20(%)	GC content(%)
CG1	60735146	60356114	98.84	46.35
CG2	63986688	63595314	98.83	47.00
CG3	63091136	62681460	98.77	46.49
TG1	60642694	60260502	98.83	46.68
TG2	68729898	68237076	98.8	46.84
TG3	63592658	63210476	98.91	46.7

Table S2. Statistics of the sequencing and assembly data.

Type	Total transcripts num	Total unigenes num	Total sequence base	Largest Length	Smallest Length	Average length	N50	GC percent	Mean mapped reads
Resource	1101680	533674	686462203	17234	201	623.1	849	42.78	159.7172405

Table S3. The annotated gene number in different databases.

Total Unigenes	Nr	KEGG	Swissport	KOG	GO	Annoted	Pfam
380155	125738	74201	96930	20572	13933	133326	67293

Table S4. The detail changes of DEGs between CG and TG group.

Id	CG_tpm	TG_tpm	log2fc	pvalue	padjust	regulate
TRINITY_DN127766_c0_g1	4.723333	0	-2.90987	1.35E-05	0.006765	down
TRINITY_DN135246_c0_g1	2.716667	1.016667	-1.28269	0.000165	0.043654	down
TRINITY_DN135764_c0_g1	0.613333	0	-2.96024	9.48E-06	0.005198	down
TRINITY_DN136637_c0_g1	5.1	1.673333	-1.76924	2.71E-06	0.001864	down
TRINITY_DN136671_c0_g1	6.74	0.096667	-3.12396	1.35E-06	0.001049	down
TRINITY_DN136866_c0_g1	1.353333	0.033333	-3.00107	2.56E-06	0.001803	down
TRINITY_DN136947_c0_g1	1.4	0.116667	-2.20585	0.000135	0.038419	down
TRINITY_DN138163_c0_g1	0.903333	0	-2.93534	8.78E-06	0.004924	down
TRINITY_DN139098_c0_g1	0.603333	0	-2.61769	9.34E-05	0.029819	down
TRINITY_DN141073_c0_g1	0.466667	0	-2.50992	0.00018	0.04653	down
TRINITY_DN141783_c3_g3	136.78	52.68667	-1.36448	9.51E-05	0.030142	down
TRINITY_DN142085_c0_g1	0.67	0.143333	-2.29712	0.000131	0.03757	down
TRINITY_DN142612_c0_g1	0.513333	0	-2.51096	0.000178	0.046491	down
TRINITY_DN142829_c1_g2	11.85667	0.946667	-2.99332	2.83E-08	3.98E-05	down
TRINITY_DN143058_c1_g2	6.946667	0	-3.58649	5.33E-08	6.60E-05	down
TRINITY_DN143122_c1_g1	3	0.626667	-1.20604	3.49E-05	0.014416	down
TRINITY_DN143375_c1_g1	2.176667	0.876667	-1.41681	0.000155	0.042066	down
TRINITY_DN143776_c0_g1	10.57667	0	-2.91965	1.30E-05	0.006607	down
TRINITY_DN144056_c0_g1	1.243333	0.016667	-3.69094	3.50E-09	6.56E-06	down
TRINITY_DN144733_c5_g2	4.95	0.36	-2.83282	2.64E-06	0.00185	down
TRINITY_DN145003_c2_g1	10.69667	5.593333	-1.18908	1.81E-06	0.001333	down
TRINITY_DN145264_c0_g1	5.903333	0.036667	-2.54916	0.000142	0.039782	down
TRINITY_DN145570_c2_g1	6.436667	0.45	-2.68826	3.10E-08	4.27E-05	down
TRINITY_DN145992_c0_g1	41.99	2.45	-2.96794	6.94E-07	0.000605	down

TRINITY_DN146340_c3_g1	0.566667	0.01	-2.7712	2.84E-05	0.012133	down
TRINITY_DN146513_c3_g2	0.843333	0.016667	-2.59869	9.55E-05	0.030171	down
TRINITY_DN146682_c0_g1	1.46	0.23	-2.03045	0.000146	0.040283	down
TRINITY_DN146788_c0_g1	1.483333	0.226667	-2.26221	6.50E-05	0.022936	down
TRINITY_DN147645_c2_g1	95.62333	14.82667	-2.41769	5.04E-08	6.37E-05	down
TRINITY_DN148505_c1_g1	1.086667	0.013333	-2.62901	8.63E-05	0.02825	down
TRINITY_DN148735_c1_g3	0.443333	0.003333	-2.92868	1.03E-05	0.005554	down
TRINITY_DN149186_c1_g2	103.04	21.61	-2.32819	1.14E-08	1.82E-05	down
TRINITY_DN150753_c0_g1	3.01	1.726667	-1.04161	9.91E-05	0.030842	down
TRINITY_DN150873_c4_g1	6.656667	0.996667	-2.22363	5.27E-05	0.019728	down
TRINITY_DN151847_c5_g1	2.473333	0.013333	-2.47126	0.000198	0.049646	down
TRINITY_DN152668_c0_g1	1.356667	0.26	-1.82239	1.58E-05	0.007571	down
TRINITY_DN152809_c0_g7	0.956667	0.17	-1.94929	4.92E-05	0.018566	down
TRINITY_DN153700_c3_g2	48.05	4.7	-3.31442	4.85E-10	1.19E-06	down
TRINITY_DN154349_c4_g1	10.35667	0.096667	-3.44064	1.66E-07	0.000179	down
TRINITY_DN154776_c0_g1	0.613333	0.103333	-1.9453	0.000161	0.042981	down
TRINITY_DN155229_c0_g1	1.243333	0.086667	-2.89226	1.64E-06	0.00124	down
TRINITY_DN155825_c3_g2	5.206667	1.096667	-1.6449	2.72E-05	0.01175	down
TRINITY_DN156063_c1_g1	11.35333	3.053333	-1.50907	7.71E-06	0.004432	down
TRINITY_DN158068_c0_g1	4.883333	0.286667	-2.71195	1.17E-05	0.006123	down
TRINITY_DN158479_c7_g1	52.95	19.29	-1.37624	2.68E-06	0.001861	down
TRINITY_DN158804_c1_g1	2.323333	0.37	-2.46225	1.72E-10	4.74E-07	down
TRINITY_DN158982_c0_g2	1.116667	0.03	-3.15502	3.62E-08	4.77E-05	down
TRINITY_DN159112_c0_g1	1.25	0.24	-1.81386	7.81E-05	0.026281	down
TRINITY_DN159438_c5_g1	2.446667	1.146667	-1.2692	8.80E-05	0.028524	down
TRINITY_DN159487_c1_g1	5.33	1.26	-1.83069	3.77E-05	0.015304	down
TRINITY_DN159636_c1_g3	24.57333	9.636667	-1.42972	5.22E-05	0.019598	down
TRINITY_DN159995_c0_g2	3.113333	0	-2.94621	1.01E-05	0.005473	down
TRINITY_DN160066_c2_g1	5.98	2.82	-1.22706	9.05E-05	0.029133	down
TRINITY_DN160207_c4_g1	19.99333	9.93	-1.16812	3.04E-06	0.002046	down
TRINITY_DN160555_c2_g2	0.456667	0.016667	-3.24089	1.41E-07	0.000155	down
TRINITY_DN161148_c5_g1	0.37	0.006667	-2.68919	3.84E-05	0.015526	down
TRINITY_DN161367_c4_g1	3.896667	2.51	-1.45415	7.97E-05	0.026699	down
TRINITY_DN161379_c1_g1	4.213333	0.276667	-2.93021	4.57E-07	0.000439	down
TRINITY_DN161667_c2_g1	2.89	1.03	-1.51119	0.000135	0.038412	down
TRINITY_DN161987_c0_g3	5.013333	0.676667	-2.355	4.80E-05	0.018256	down
TRINITY_DN162960_c0_g3	5.013333	1.58	-1.22309	0.000128	0.037068	down
TRINITY_DN163030_c0_g1	13.08333	2.82	-2.07501	6.13E-08	7.45E-05	down
TRINITY_DN163377_c2_g1	9.516667	3.673333	-1.42487	1.20E-06	0.000961	down
TRINITY_DN163479_c2_g1	2.13	1.24	-2.50361	1.35E-05	0.006767	down
TRINITY_DN163876_c0_g2	0.593333	0.07	-2.2088	0.000161	0.042981	down
TRINITY_DN163890_c2_g3	0.523333	0	-3.25813	7.02E-07	0.000608	down
TRINITY_DN163893_c2_g4	2.62	0.443333	-1.86485	2.02E-05	0.00925	down
TRINITY_DN164081_c0_g1	2.41	0.333333	-2.18211	1.05E-05	0.005681	down
TRINITY_DN164115_c0_g1	264.7367	85.55667	-1.98685	7.97E-10	1.80E-06	down
TRINITY_DN164129_c1_g1	35.11667	17.41333	-1.08733	3.26E-08	4.44E-05	down
TRINITY_DN165279_c0_g1	0.83	0.006667	-3.24076	8.29E-07	0.000699	down
TRINITY_DN165552_c1_g1	2.833333	0.72	-1.48271	0.000142	0.039828	down
TRINITY_DN165723_c1_g1	3.083333	2.32	-2.3682	0.000103	0.03166	down
TRINITY_DN167312_c0_g1	5.78	2.35	-1.39447	1.71E-05	0.008031	down
TRINITY_DN169240_c0_g2	2.07	0.203333	-1.80337	7.65E-05	0.025903	down
TRINITY_DN169375_c2_g2	3.113333	0.69	-1.5402	3.70E-05	0.01509	down

TRINITY_DN169622_c2_g3	2.22	0.16	-2.99122	1.79E-08	2.64E-05	down
TRINITY_DN169733_c1_g3	1.286667	0	-2.66419	6.99E-05	0.024267	down
TRINITY_DN170233_c3_g1	2.953333	0.083333	-2.55589	4.68E-05	0.017946	down
TRINITY_DN170906_c0_g1	4.356667	2.32	-1.34759	0.000158	0.042454	down
TRINITY_DN171136_c0_g2	300.9367	127.37	-1.28391	2.79E-05	0.011943	down
TRINITY_DN171554_c0_g1	1.74	0.093333	-2.24615	7.37E-05	0.025221	down
TRINITY_DN172031_c0_g1	1.836667	0.42	-1.74377	8.85E-07	0.000726	down
TRINITY_DN172138_c0_g1	3.633333	1.42	-1.40938	0.000141	0.039663	down
TRINITY_DN172836_c3_g1	6.916667	2.333333	-1.46595	3.34E-06	0.002215	down
TRINITY_DN173226_c1_g1	3.4	0.03	-2.78006	3.29E-05	0.013773	down
TRINITY_DN173527_c3_g3	0.856667	0.1	-2.41815	2.74E-05	0.011769	down
TRINITY_DN173599_c3_g1	4.033333	1.673333	-1.329	0.000114	0.034114	down
TRINITY_DN173708_c1_g1	1.216667	0.283333	-2.22887	5.86E-05	0.021352	down
TRINITY_DN173921_c2_g2	0.973333	0.156667	-2.32583	2.18E-06	0.001564	down
TRINITY_DN174228_c1_g1	1.036667	0.19	-1.79568	0.000143	0.039938	down
TRINITY_DN175240_c3_g1	9.016667	3.413333	-1.40019	0.000155	0.042066	down
TRINITY_DN175558_c2_g1	195.6	23.78333	-2.29471	5.85E-09	1.04E-05	down
TRINITY_DN175862_c1_g5	2.206667	1.003333	-1.08223	2.61E-05	0.011505	down
TRINITY_DN176773_c1_g1	15.91667	4.933333	-1.60112	8.61E-05	0.02825	down
TRINITY_DN177026_c0_g1	6.526667	0.85	-2.86322	8.75E-14	4.52E-10	down
TRINITY_DN177191_c3_g1	0.636667	0	-2.68834	5.92E-05	0.02143	down
TRINITY_DN177292_c2_g2	0.7	0.03	-2.62175	4.26E-05	0.01665	down
TRINITY_DN177755_c0_g1	2.196667	0.21	-3.08813	6.66E-07	0.000585	down
TRINITY_DN177757_c1_g1	0.87	0.263333	-1.60431	0.000104	0.031903	down
TRINITY_DN178457_c0_g1	17.87333	9.983333	-1.12862	0.000103	0.03166	down
TRINITY_DN178541_c0_g1	3.756667	0.596667	-2.16341	0.000138	0.039119	down
TRINITY_DN178616_c1_g2	4.17	0.6	-2.15688	0.0002	0.049739	down
TRINITY_DN178690_c0_g1	2.736667	0.833333	-1.65625	1.34E-05	0.006731	down
TRINITY_DN178815_c0_g1	36.28333	16.97	-1.1781	7.39E-05	0.025221	down
TRINITY_DN179493_c1_g1	4.073333	0.523333	-2.14002	1.81E-05	0.008385	down
TRINITY_DN179545_c7_g1	1.923333	0.106667	-2.85906	2.76E-06	0.00189	down
TRINITY_DN179971_c0_g1	329.69	132.9467	-1.3651	7.89E-05	0.02648	down
TRINITY_DN180283_c2_g1	1.336667	0.223333	-2.13185	7.79E-05	0.026281	down
TRINITY_DN180393_c1_g1	7.196667	2.243333	-1.54548	0.000185	0.047437	down
TRINITY_DN180793_c1_g1	12.08333	4.116667	-1.40006	5.04E-06	0.003104	down
TRINITY_DN180968_c0_g1	15.57667	5.353333	-1.76421	7.28E-09	1.23E-05	down
TRINITY_DN181423_c1_g2	3.113333	0.113333	-2.82268	1.81E-05	0.008378	down
TRINITY_DN181459_c0_g1	1.406667	0.466667	-1.48138	8.67E-05	0.02825	down
TRINITY_DN181529_c2_g2	6.153333	2.776667	-1.33151	0.000191	0.048591	down
TRINITY_DN181859_c0_g1	2.03	0.013333	-6.46461	1.94E-45	2.40E-40	down
TRINITY_DN182309_c1_g2	22.45333	5.876667	-1.79851	4.55E-05	0.017611	down
TRINITY_DN182357_c1_g1	27.76	11.24667	-1.45971	1.45E-06	0.001111	down
TRINITY_DN182513_c0_g1	6.466667	0.8	-2.49651	7.62E-08	8.99E-05	down
TRINITY_DN182748_c1_g1	0.786667	0.176667	-1.96197	3.20E-07	0.000322	down
TRINITY_DN182759_c2_g2	6.363333	3.863333	-1.95215	0.000183	0.047167	down
TRINITY_DN182842_c0_g1	5.97	0.84	-2.39012	1.51E-06	0.001145	down
TRINITY_DN183168_c3_g1	1.573333	0.14	-2.66307	4.58E-06	0.002864	down
TRINITY_DN183175_c2_g4	1.353333	0	-2.50216	0.000187	0.047957	down
TRINITY_DN183191_c0_g2	4.223333	1.636667	-1.41184	3.24E-05	0.013646	down
TRINITY_DN183252_c4_g3	0.506667	0	-2.80871	2.67E-05	0.011725	down
TRINITY_DN183294_c2_g1	48.59333	13.21333	-2.04537	2.17E-05	0.009832	down
TRINITY_DN183336_c1_g1	20.64	3.17	-2.31415	4.23E-05	0.01665	down

TRINITY_DN184358_c1_g4	2.543333	0.433333	-2.15366	5.06E-05	0.019064	down
TRINITY_DN184649_c0_g1	2.286667	0.763333	-1.60141	7.60E-05	0.025801	down
TRINITY_DN184934_c1_g1	6.77	2.203333	-1.50115	3.66E-05	0.014972	down
TRINITY_DN185257_c0_g2	9.8	3.216667	-1.72647	6.07E-10	1.42E-06	down
TRINITY_DN185373_c0_g1	2.43	1.05	-1.46859	3.44E-05	0.014291	down
TRINITY_DN185422_c1_g2	7.113333	3.173333	-1.09482	6.51E-06	0.003934	down
TRINITY_DN185826_c0_g2	5.736667	2.563333	-1.08266	4.22E-05	0.016648	down
TRINITY_DN186429_c2_g1	17.42333	2.573333	-2.27466	2.69E-05	0.011725	down
TRINITY_DN187255_c1_g1	76.58667	24.02333	-1.7271	8.84E-07	0.000726	down
TRINITY_DN188848_c2_g1	18.40667	9.41	-1.01022	1.68E-05	0.00795	down
TRINITY_DN189350_c2_g1	16.29	7.98	-1.02217	1.40E-07	0.000155	down
TRINITY_DN189421_c1_g2	1.403333	0.286667	-1.8471	1.27E-06	0.00101	down
TRINITY_DN190168_c0_g1	7.07	2.916667	-1.37415	0.000179	0.04653	down
TRINITY_DN191128_c2_g1	26.81667	10.83667	-1.03022	2.96E-09	5.74E-06	down
TRINITY_DN191861_c4_g3	8.95	1.456667	-2.35417	3.92E-06	0.002504	down
TRINITY_DN191924_c1_g2	1.203333	0.053333	-2.78214	8.53E-06	0.004826	down
TRINITY_DN192433_c3_g1	0.826667	1.27	-1.91965	1.21E-05	0.006296	down
TRINITY_DN192604_c1_g1	392.3867	201.3633	-1.13129	7.41E-07	0.000633	down
TRINITY_DN192944_c0_g1	14.86	6.623333	-1.15823	3.20E-06	0.002134	down
TRINITY_DN193239_c4_g3	13.89667	4.46	-1.69196	3.25E-07	0.000325	down
TRINITY_DN193848_c4_g2	7.53	1.196667	-2.46305	1.32E-09	2.77E-06	down
TRINITY_DN194849_c3_g2	2.086667	1.013333	-1.17636	0.000153	0.041657	down
TRINITY_DN195061_c8_g2	5.9	1.49	-1.86844	0.000113	0.03393	down
TRINITY_DN195653_c6_g4	117.6567	21.82667	-2.35848	9.96E-19	9.50E-15	down
TRINITY_DN196487_c16_g1	1.303333	0.07	-3.50464	8.46E-11	2.69E-07	down
TRINITY_DN100825_c0_g1	0	0.236667	2.617143	8.80E-05	0.028524	up
TRINITY_DN118163_c0_g2	0	0.24	2.823309	2.06E-05	0.009364	up
TRINITY_DN124702_c0_g1	0.013333	0.426667	2.590749	6.76E-05	0.023582	up
TRINITY_DN126667_c0_g2	0	0.223333	3.062096	2.95E-06	0.002	up
TRINITY_DN133098_c0_g1	0.046667	0.306667	2.138415	0.000144	0.039938	up
TRINITY_DN134102_c0_g1	7.353333	17.40667	1.07133	2.69E-06	0.001861	up
TRINITY_DN134618_c0_g2	0.103333	1.176667	2.544587	1.47E-05	0.007184	up
TRINITY_DN136604_c0_g1	0	0.72	2.750802	3.96E-05	0.015806	up
TRINITY_DN137611_c0_g2	0.003333	0.233333	3.037068	1.33E-06	0.00104	up
TRINITY_DN138741_c0_g1	14.78333	87.66	2.168248	2.07E-12	8.28E-09	up
TRINITY_DN139673_c1_g1	2.243333	13.64333	2.467127	3.30E-14	1.78E-10	up
TRINITY_DN140382_c0_g3	0.02	0.346667	2.416358	0.000165	0.043654	up
TRINITY_DN140556_c2_g1	2.566667	20.82333	2.520987	1.13E-07	0.000127	up
TRINITY_DN140647_c0_g1	9.983333	27.64333	1.429351	3.99E-07	0.000392	up
TRINITY_DN142199_c0_g1	0.06	1.103333	2.803453	3.95E-06	0.002507	up
TRINITY_DN142443_c8_g1	1.72	7.666667	4.352226	2.13E-23	5.27E-19	up
TRINITY_DN143569_c0_g1	0.55	2.653333	1.86362	9.86E-05	0.030774	up
TRINITY_DN143702_c0_g3	2.87	20.92333	2.095639	5.41E-05	0.020186	up
TRINITY_DN143762_c4_g1	0.026667	3.973333	5.273245	5.39E-22	9.54E-18	up
TRINITY_DN145745_c0_g1	0.046667	3.736667	2.537868	0.00015	0.041118	up
TRINITY_DN146435_c0_g2	0.106667	2.3	2.83215	3.47E-06	0.002265	up
TRINITY_DN146633_c0_g1	0	3.006667	2.917298	1.08E-05	0.005803	up
TRINITY_DN147072_c0_g2	7.353333	20.85333	1.297613	0.000143	0.039938	up
TRINITY_DN147219_c3_g1	0	14.89667	6.642685	1.32E-32	8.17E-28	up
TRINITY_DN147309_c6_g3	0.173333	0.96	2.273275	0.000116	0.034656	up
TRINITY_DN147908_c0_g3	0.196667	1.746667	2.227713	0.000153	0.041673	up
TRINITY_DN147984_c5_g1	0.216667	2.573333	2.571652	9.30E-06	0.005118	up

TRINITY_DN148042_c5_g1	0.086667	0.95	2.333162	0.000124	0.036105	up
TRINITY_DN148154_c0_g1	0	0.613333	3.612113	1.78E-08	2.64E-05	up
TRINITY_DN148250_c3_g8	7.65	156.0367	2.544885	9.05E-05	0.029133	up
TRINITY_DN148360_c3_g1	0.566667	0.87	1.909985	6.27E-05	0.022372	up
TRINITY_DN148821_c0_g8	0.11	1.353333	2.608513	8.86E-06	0.004944	up
TRINITY_DN149276_c9_g2	0	0.923333	2.614468	8.58E-05	0.02825	up
TRINITY_DN149281_c3_g1	0.05	1.756667	3.47644	3.16E-09	6.03E-06	up
TRINITY_DN149434_c0_g2	0.456667	1.51	1.806539	9.65E-05	0.030335	up
TRINITY_DN149825_c2_g3	0	0.216667	2.838389	1.76E-05	0.00825	up
TRINITY_DN149847_c5_g2	0	3.826667	5.274515	8.29E-20	9.33E-16	up
TRINITY_DN149852_c0_g1	0.466667	1.74	1.526174	4.73E-05	0.018097	up
TRINITY_DN150473_c0_g2	0	24.25333	2.943599	7.73E-06	0.004432	up
TRINITY_DN150538_c14_g2	0.02	0.75	2.788374	1.24E-05	0.006357	up
TRINITY_DN150701_c1_g1	2.936667	75.69667	2.616836	5.84E-05	0.021352	up
TRINITY_DN150718_c5_g2	0.35	1.433333	1.638719	0.000141	0.039663	up
TRINITY_DN150917_c0_g1	0.173333	2.46	2.540789	5.63E-05	0.020899	up
TRINITY_DN150928_c4_g1	0.043333	0.56	2.413787	7.48E-05	0.02546	up
TRINITY_DN151394_c12_g1	0.176667	1.973333	2.451946	4.08E-05	0.016264	up
TRINITY_DN151523_c5_g2	0.02	0.446667	2.796526	1.70E-05	0.007997	up
TRINITY_DN151523_c5_g4	0	0.2	2.60003	9.62E-05	0.030327	up
TRINITY_DN151705_c0_g1	2.03	3.896667	1.28019	8.10E-05	0.027048	up
TRINITY_DN151807_c4_g1	1.006667	2.82	1.909903	5.71E-07	0.00052	up
TRINITY_DN152008_c0_g1	12.76667	45.57	1.614985	9.51E-05	0.030142	up
TRINITY_DN152013_c6_g1	0.146667	3.02	3.704954	1.20E-16	9.30E-13	up
TRINITY_DN152361_c5_g1	0.063333	1.073333	2.551145	0.000123	0.036102	up
TRINITY_DN153311_c0_g2	0	0.61	3.148291	1.39E-06	0.00107	up
TRINITY_DN153388_c4_g1	0.34	5.533333	2.526424	4.66E-05	0.01792	up
TRINITY_DN153512_c1_g1	0.12	0.713333	2.328721	3.51E-05	0.014465	up
TRINITY_DN154555_c8_g2	0.08	0.863333	2.445489	2.68E-05	0.011725	up
TRINITY_DN154691_c3_g1	0.006667	0.243333	2.713732	2.50E-05	0.011083	up
TRINITY_DN154777_c3_g5	0.06	1.236667	2.432941	0.000176	0.046244	up
TRINITY_DN154832_c1_g1	1.076667	3.87	1.533845	0.000125	0.036382	up
TRINITY_DN154889_c5_g2	0	3.03	3.716885	3.64E-09	6.73E-06	up
TRINITY_DN155203_c11_g1	0.5	6.88	4.369127	1.52E-22	3.15E-18	up
TRINITY_DN155482_c1_g1	0.006667	0.47	2.856273	1.37E-05	0.006801	up
TRINITY_DN155675_c4_g1	0	0.64	4.021164	1.08E-10	3.20E-07	up
TRINITY_DN155709_c5_g1	0	2.043333	3.447817	7.69E-08	8.99E-05	up
TRINITY_DN155733_c2_g2	0.153333	2.886667	2.643552	2.04E-05	0.009345	up
TRINITY_DN155955_c0_g3	0.013333	0.536667	2.940871	3.47E-06	0.002265	up
TRINITY_DN155978_c2_g11	0	10.6	2.792534	2.57E-05	0.011385	up
TRINITY_DN156018_c1_g3	2.326667	5.9	2.467124	1.97E-13	9.37E-10	up
TRINITY_DN156101_c1_g3	12.08	34.40667	1.216145	8.60E-05	0.02825	up
TRINITY_DN156399_c1_g2	0.033333	1.276667	2.746017	1.88E-05	0.008648	up
TRINITY_DN156404_c3_g6	0	1.6	3.388291	1.78E-07	0.000189	up
TRINITY_DN156591_c4_g2	0.626667	1.8	2.994102	1.19E-07	0.000133	up
TRINITY_DN156812_c3_g1	0.03	3.216667	3.89426	6.86E-10	1.57E-06	up
TRINITY_DN156821_c0_g1	0	0.473333	2.554611	0.00013	0.03757	up
TRINITY_DN156826_c3_g2	0.183333	2.876667	2.478225	0.000123	0.036105	up
TRINITY_DN157137_c5_g1	6.183333	20.72667	1.579262	4.38E-15	2.86E-11	up
TRINITY_DN157184_c2_g1	0.05	0.91	2.723115	1.23E-05	0.006324	up
TRINITY_DN157234_c2_g3	4.51	15.09667	1.599263	4.23E-07	0.00041	up
TRINITY_DN157480_c3_g3	0.26	4.93	2.86761	1.18E-06	0.00095	up

TRINITY_DN157480_c5_g1	1.33	8.44	2.650295	8.89E-11	2.75E-07	up
TRINITY_DN157565_c0_g2	1.673333	7.313333	1.745285	0.000144	0.040001	up
TRINITY_DN157577_c0_g1	0.303333	1.086667	1.625485	3.85E-05	0.015526	up
TRINITY_DN157992_c5_g4	0	1.22	3.89171	4.23E-10	1.07E-06	up
TRINITY_DN157997_c4_g1	0.03	3.666667	4.625563	4.23E-16	3.08E-12	up
TRINITY_DN158203_c2_g2	0	1.643333	2.894101	1.38E-05	0.006803	up
TRINITY_DN158222_c1_g5	0.016667	0.456667	2.863723	4.73E-06	0.002943	up
TRINITY_DN158331_c6_g2	0.036667	0.666667	2.629117	2.17E-05	0.009832	up
TRINITY_DN158993_c4_g6	0.033333	1.836667	3.016248	2.39E-06	0.001702	up
TRINITY_DN159078_c1_g1	2.086667	3.113333	1.628361	9.17E-05	0.029415	up
TRINITY_DN159531_c3_g2	0	1.766667	3.558866	2.55E-08	3.63E-05	up
TRINITY_DN159532_c7_g1	0.083333	1.05	2.493195	2.71E-05	0.011725	up
TRINITY_DN159691_c5_g5	0.466667	6.276667	2.741035	2.47E-05	0.010986	up
TRINITY_DN159959_c5_g3	0.043333	14.17667	2.937617	1.13E-05	0.006003	up
TRINITY_DN159981_c5_g1	0	1.85	4.886027	1.20E-16	9.30E-13	up
TRINITY_DN160157_c0_g2	0.26	3.653333	2.632243	1.30E-05	0.006607	up
TRINITY_DN160215_c3_g2	0.03	0.996667	3.033366	9.97E-07	0.000812	up
TRINITY_DN160441_c7_g1	0.083333	1.806667	2.799275	4.85E-06	0.003007	up
TRINITY_DN160538_c3_g2	0	9.493333	3.593669	1.64E-08	2.51E-05	up
TRINITY_DN160557_c2_g1	0.01	0.87	2.681291	6.10E-05	0.021853	up
TRINITY_DN160677_c4_g1	0	0.58	2.58792	0.000105	0.032098	up
TRINITY_DN160939_c4_g1	0.13	1.493333	2.581353	6.56E-06	0.003934	up
TRINITY_DN161250_c5_g2	0.013333	0.306667	2.970715	1.69E-06	0.001253	up
TRINITY_DN161325_c2_g1	0.016667	3.633333	2.497515	0.000185	0.047437	up
TRINITY_DN161496_c2_g6	0.013333	1.936667	4.611013	5.73E-16	3.95E-12	up
TRINITY_DN161590_c2_g3	0.07	2.546667	3.105402	6.25E-07	0.000553	up
TRINITY_DN161596_c0_g1	0	30.72667	3.184858	1.18E-06	0.00095	up
TRINITY_DN161873_c2_g2	0.043333	0.816667	2.709108	1.16E-05	0.006091	up
TRINITY_DN161915_c1_g4	4.673333	8.633333	1.200476	0.000101	0.031148	up
TRINITY_DN162583_c0_g1	0.03	1.636667	2.25417	0.000182	0.047031	up
TRINITY_DN162721_c0_g8	0.006667	0.273333	3.019575	1.66E-06	0.00124	up
TRINITY_DN162850_c1_g1	0.023333	0.806667	2.586535	8.65E-05	0.02825	up
TRINITY_DN162867_c3_g1	5.693333	22.41	1.829715	6.80E-15	4.21E-11	up
TRINITY_DN163161_c1_g2	0.936667	1.84	2.506712	4.29E-05	0.016698	up
TRINITY_DN163306_c1_g1	2.03	3.19	1.825364	1.58E-05	0.007571	up
TRINITY_DN163853_c1_g1	0.03	0.356667	2.389402	8.24E-05	0.027281	up
TRINITY_DN164095_c2_g1	0	1.19	2.537003	0.00014	0.039584	up
TRINITY_DN164196_c5_g2	0.12	0.756667	2.028544	6.88E-05	0.023941	up
TRINITY_DN164219_c0_g2	0.7	3.476667	2.108461	3.74E-06	0.002412	up
TRINITY_DN164228_c3_g2	0	2.986667	3.598933	1.66E-08	2.51E-05	up
TRINITY_DN164298_c4_g1	0.03	0.863333	2.844393	8.13E-06	0.004618	up
TRINITY_DN164502_c0_g1	0.156667	2.293333	2.718049	4.12E-06	0.002603	up
TRINITY_DN164555_c2_g1	0.133333	2.63	2.85561	1.35E-06	0.001049	up
TRINITY_DN164561_c2_g1	3.926667	12.79	1.54211	1.84E-06	0.00135	up
TRINITY_DN164708_c2_g3	0.04	1.703333	3.2644	1.53E-07	0.000167	up
TRINITY_DN164801_c0_g2	0.62	4.543333	2.451348	7.44E-09	1.23E-05	up
TRINITY_DN164802_c1_g1	0.15	1.166667	2.266014	9.20E-06	0.005101	up
TRINITY_DN164809_c2_g1	0.133333	2.516667	2.360665	0.000193	0.04881	up
TRINITY_DN165098_c0_g1	123.63	376.03	1.311329	6.76E-05	0.023582	up
TRINITY_DN165124_c7_g1	0	1.493333	4.230907	1.35E-11	5.22E-08	up
TRINITY_DN165176_c1_g3	0.346667	4.12	2.382037	0.000191	0.048591	up
TRINITY_DN165329_c8_g3	0.083333	1.16	2.640229	1.17E-05	0.006123	up

TRINITY_DN165369_c3_g2	0.006667	0.993333	4.312804	6.14E-13	2.54E-09	up
TRINITY_DN165640_c2_g1	0	0.55	2.732608	4.33E-05	0.016806	up
TRINITY_DN165658_c6_g1	0	0.223333	2.486018	0.000199	0.049646	up
TRINITY_DN165757_c0_g1	0.38	1.326667	2.796034	4.56E-11	1.53E-07	up
TRINITY_DN165844_c0_g2	0.01	0.563333	3.137179	5.28E-07	0.000484	up
TRINITY_DN166050_c0_g1	1.853333	4.646667	1.208679	0.000134	0.038288	up
TRINITY_DN166156_c7_g1	0.15	0.81	1.988036	0.000172	0.04541	up
TRINITY_DN166219_c3_g3	0.033333	1.39	3.668252	1.48E-10	4.21E-07	up
TRINITY_DN166361_c5_g2	0	0.846667	3.38216	1.98E-07	0.000206	up
TRINITY_DN166362_c4_g4	0	1.73	3.367229	1.69E-07	0.00018	up
TRINITY_DN166570_c1_g1	0.05	2.156667	3.409617	5.49E-08	6.73E-05	up
TRINITY_DN166629_c4_g1	0	1.943333	2.945154	7.27E-06	0.004308	up
TRINITY_DN166639_c1_g2	0.026667	0.883333	3.818519	1.48E-11	5.41E-08	up
TRINITY_DN166667_c1_g6	0	0.85	3.83464	1.57E-09	3.23E-06	up
TRINITY_DN167488_c4_g1	0.003333	2.973333	3.809536	1.82E-10	4.90E-07	up
TRINITY_DN167523_c2_g1	0.106667	13.81667	4.913552	4.13E-21	6.39E-17	up
TRINITY_DN167719_c1_g1	0.336667	79.64333	2.530189	0.000158	0.042454	up
TRINITY_DN167803_c3_g1	6.533333	20.64	1.35222	2.12E-06	0.001537	up
TRINITY_DN167832_c5_g1	0.27	3.893333	2.504772	0.00016	0.042881	up
TRINITY_DN167931_c3_g3	0.05	0.44	2.448181	0.000126	0.036572	up
TRINITY_DN168201_c0_g2	0	7.073333	2.701249	4.85E-05	0.018358	up
TRINITY_DN168423_c2_g1	2.05	57.29667	2.521573	0.000147	0.040602	up
TRINITY_DN168758_c0_g5	0.036667	0.98	2.814103	7.67E-06	0.004432	up
TRINITY_DN168971_c2_g3	0.013333	0.61	2.88411	6.41E-06	0.00389	up
TRINITY_DN169017_c0_g3	2.243333	8.506667	1.599686	3.15E-06	0.002109	up
TRINITY_DN169104_c0_g2	0.206667	1.85	2.296123	7.25E-05	0.025027	up
TRINITY_DN169409_c3_g5	0	7.196667	3.500967	4.49E-08	5.73E-05	up
TRINITY_DN169425_c4_g1	0.023333	0.363333	2.36674	0.000179	0.046491	up
TRINITY_DN169463_c3_g3	0.833333	3.993333	1.90563	5.81E-06	0.003548	up
TRINITY_DN169874_c0_g2	0.703333	0.87	1.445463	0.000177	0.046244	up
TRINITY_DN170062_c0_g1	0	0.246667	2.500921	0.000183	0.047256	up
TRINITY_DN170134_c6_g2	0.01	0.603333	3.15595	4.85E-07	0.000452	up
TRINITY_DN170134_c6_g5	0	0.63	2.560508	0.000128	0.037068	up
TRINITY_DN170367_c4_g1	0.106667	1.056667	2.680078	2.86E-08	3.99E-05	up
TRINITY_DN170423_c0_g1	0	1.453333	3.2948	5.75E-07	0.00052	up
TRINITY_DN170524_c5_g1	5.216667	5.93	3.251004	9.96E-09	1.62E-05	up
TRINITY_DN170632_c0_g1	0.81	49.88333	3.28078	3.81E-07	0.000378	up
TRINITY_DN170866_c2_g2	0.17	0.986667	2.014292	6.73E-06	0.00401	up
TRINITY_DN171212_c4_g1	0	0.64	3.546549	4.01E-08	5.23E-05	up
TRINITY_DN171303_c3_g2	2.436667	197.1033	2.57499	0.000121	0.035717	up
TRINITY_DN171307_c3_g4	0.02	0.863333	2.817897	1.78E-05	0.008278	up
TRINITY_DN171876_c5_g3	0.023333	1.036667	3.179239	4.21E-07	0.00041	up
TRINITY_DN171936_c0_g1	6.856667	8.26	1.569056	3.59E-05	0.014709	up
TRINITY_DN171955_c1_g1	0.86	46.31	2.710247	4.75E-05	0.018105	up
TRINITY_DN171982_c0_g1	0.076667	1.843333	2.780548	9.22E-06	0.005101	up
TRINITY_DN172016_c4_g2	0.246667	1.243333	2.040626	8.13E-05	0.027063	up
TRINITY_DN172044_c1_g1	0.073333	0.713333	2.253845	0.000136	0.038604	up
TRINITY_DN172164_c3_g1	0.01	0.296667	2.596	6.43E-05	0.022771	up
TRINITY_DN172249_c5_g2	0.003333	0.36	4.036374	3.20E-11	1.10E-07	up
TRINITY_DN172321_c2_g2	12.09667	27.45333	1.060586	5.66E-06	0.00347	up
TRINITY_DN172375_c0_g1	0	0.34	4.02512	1.50E-10	4.21E-07	up
TRINITY_DN172722_c2_g3	0	0.506667	2.642455	7.35E-05	0.025221	up

TRINITY_DN172727_c1_g1	1.09	9.213333	2.281671	8.23E-05	0.027281	up
TRINITY_DN172753_c3_g4	0.01	0.303333	3.11092	8.13E-08	9.41E-05	up
TRINITY_DN173291_c0_g1	0.18	1.563333	2.448171	2.45E-06	0.001736	up
TRINITY_DN173358_c4_g1	0.143333	3.873333	3.286134	2.29E-08	3.34E-05	up
TRINITY_DN173385_c1_g3	0.96	3.67	1.933684	4.31E-08	5.57E-05	up
TRINITY_DN173730_c6_g1	0	0.916667	2.891855	1.18E-05	0.006159	up
TRINITY_DN173919_c4_g1	0.036667	3.07	3.868534	1.02E-10	3.09E-07	up
TRINITY_DN173943_c0_g3	0.256667	1.346667	1.890473	0.000148	0.040602	up
TRINITY_DN174094_c1_g1	0	1.02	2.780891	2.88E-05	0.012245	up
TRINITY_DN174096_c1_g2	0.643333	2.153333	1.492682	4.72E-07	0.000449	up
TRINITY_DN174220_c0_g1	0.773333	6.496667	2.362019	6.58E-05	0.023146	up
TRINITY_DN174290_c1_g1	0.533333	2.656667	1.83444	0.000148	0.040602	up
TRINITY_DN174522_c0_g1	0.026667	0.51	2.634473	3.46E-05	0.014338	up
TRINITY_DN174570_c0_g1	0	5.553333	3.087278	2.17E-06	0.001564	up
TRINITY_DN174622_c2_g1	0.536667	47.80333	4.53569	2.61E-14	1.50E-10	up
TRINITY_DN174667_c2_g4	0.026667	14.32667	5.898178	1.40E-29	5.78E-25	up
TRINITY_DN174757_c1_g10	0	3.023333	3.199317	1.30E-06	0.001029	up
TRINITY_DN174896_c1_g4	0.553333	3.563333	3.243027	1.62E-09	3.29E-06	up
TRINITY_DN174928_c2_g1	0.023333	0.93	3.388319	3.61E-08	4.77E-05	up
TRINITY_DN175041_c0_g2	12.92333	30.77667	1.098999	3.54E-08	4.77E-05	up
TRINITY_DN175144_c2_g3	0.756667	3.42	1.549991	1.53E-05	0.007432	up
TRINITY_DN175184_c1_g2	0.006667	0.623333	4.173133	4.68E-13	2.00E-09	up
TRINITY_DN175251_c0_g1	0.61	5.133333	2.26882	0.000191	0.048591	up
TRINITY_DN175552_c3_g3	0.03	0.653333	2.517835	0.000124	0.036105	up
TRINITY_DN175574_c1_g1	0.02	3.416667	2.538885	0.00015	0.041118	up
TRINITY_DN175579_c3_g1	0.54	5.596667	2.337327	0.000111	0.033551	up
TRINITY_DN175610_c3_g1	0.096667	3.303333	3.268289	2.56E-07	0.000262	up
TRINITY_DN175773_c0_g1	0	1.54	3.962227	2.79E-10	7.19E-07	up
TRINITY_DN176039_c2_g2	0.053333	0.8	2.562223	3.29E-05	0.013773	up
TRINITY_DN176053_c9_g1	0	3.256667	3.752479	2.32E-09	4.64E-06	up
TRINITY_DN176370_c1_g1	0.023333	0.523333	2.648803	6.04E-05	0.021757	up
TRINITY_DN176619_c8_g1	0.63	0.616667	1.343408	0.000111	0.033624	up
TRINITY_DN176955_c4_g2	0.116667	1.653333	2.870744	1.87E-07	0.000196	up
TRINITY_DN177001_c3_g2	0.11	0.66	2.171618	0.000165	0.043654	up
TRINITY_DN177002_c0_g1	0.316667	5.17	2.729488	7.92E-06	0.00452	up
TRINITY_DN177076_c2_g2	0.04	8.123333	5.628007	1.79E-27	5.55E-23	up
TRINITY_DN177255_c0_g1	0.646667	24.03	2.569372	0.000115	0.03445	up
TRINITY_DN177368_c1_g2	0.306667	1.473333	1.898733	4.26E-05	0.01665	up
TRINITY_DN177684_c2_g2	0.03	0.596667	2.72183	7.38E-06	0.004333	up
TRINITY_DN177975_c0_g3	0.33	4.156667	2.411167	0.000112	0.033819	up
TRINITY_DN177993_c10_g3	0.093333	1.3	2.741379	1.23E-05	0.006324	up
TRINITY_DN178498_c1_g1	0.183333	0.706667	1.620303	6.42E-05	0.022771	up
TRINITY_DN179285_c2_g1	1.943333	8.863333	1.840385	6.02E-05	0.021752	up
TRINITY_DN179487_c0_g1	1.12	3.22	1.591606	2.70E-05	0.011725	up
TRINITY_DN180103_c0_g2	0.883333	2.056667	2.241142	2.38E-13	1.09E-09	up
TRINITY_DN180370_c1_g1	0	0.323333	2.676283	5.74E-05	0.021075	up
TRINITY_DN180394_c3_g1	0.036667	0.963333	2.715766	1.64E-05	0.007804	up
TRINITY_DN180665_c1_g1	0	1.086667	2.527827	0.000156	0.042088	up
TRINITY_DN180802_c2_g2	0.03	2.47	3.594567	7.17E-09	1.23E-05	up
TRINITY_DN180898_c3_g1	4.563333	4.31	3.05606	4.82E-07	0.000452	up
TRINITY_DN180994_c6_g1	0	9.743333	3.620996	1.13E-08	1.82E-05	up
TRINITY_DN181071_c1_g2	0.24	7.356667	3.400931	5.18E-09	9.31E-06	up

TRINITY_DN181159_c1_g4	2.08	97.25667	2.865505	1.55E-05	0.007507	up
TRINITY_DN181183_c0_g2	0.026667	0.46	2.661064	1.13E-05	0.006003	up
TRINITY_DN181520_c2_g1	0.15	4.606667	3.784254	1.45E-11	5.41E-08	up
TRINITY_DN181867_c3_g2	0.016667	3.286667	4.930107	1.51E-20	1.87E-16	up
TRINITY_DN182146_c0_g1	0.163333	2.763333	2.653444	8.74E-06	0.004922	up
TRINITY_DN182384_c3_g3	0	1.04	3.052691	3.47E-06	0.002265	up
TRINITY_DN182425_c1_g1	0.006667	0.546667	3.55845	7.47E-09	1.23E-05	up
TRINITY_DN182822_c2_g3	0.033333	4.7	5.024364	6.49E-21	8.93E-17	up
TRINITY_DN182872_c7_g2	0.546667	5.053333	2.28462	0.000135	0.038419	up
TRINITY_DN182951_c6_g4	0.08	1.62	2.604008	3.41E-05	0.014222	up
TRINITY_DN182959_c1_g1	0	1.93	4.602728	1.08E-13	5.36E-10	up
TRINITY_DN182964_c3_g1	0.123333	1.08	2.410303	7.12E-07	0.000612	up
TRINITY_DN182964_c3_g2	0	2.156667	2.590643	0.00011	0.033551	up
TRINITY_DN183013_c1_g1	0.366667	1.083333	1.4072	9.68E-05	0.030373	up
TRINITY_DN183104_c1_g3	0.043333	0.99	2.550876	4.28E-06	0.002694	up
TRINITY_DN183959_c1_g2	0.096667	2.17	2.389876	0.0001	0.03107	up
TRINITY_DN183964_c3_g1	0.323333	4.263333	2.477009	6.60E-05	0.023152	up
TRINITY_DN184018_c1_g1	1.716667	3.35	1.443707	9.74E-05	0.030459	up
TRINITY_DN184050_c1_g1	2.12	2.126667	2.307724	6.69E-09	1.17E-05	up
TRINITY_DN184116_c2_g3	0.36	6.08	2.730011	7.59E-06	0.004412	up
TRINITY_DN184228_c5_g1	0.06	1.196667	2.965401	7.13E-08	8.49E-05	up
TRINITY_DN184644_c2_g1	0.023333	0.653333	2.981185	8.53E-07	0.000714	up
TRINITY_DN185125_c1_g1	0.433333	1.396667	2.614597	5.58E-05	0.020759	up
TRINITY_DN185514_c0_g1	1.296667	6.51	1.263219	7.32E-06	0.004317	up
TRINITY_DN185647_c1_g3	0.036667	1.556667	2.939832	2.08E-06	0.001517	up
TRINITY_DN186424_c3_g1	0.23	7.74	2.589207	0.000111	0.033648	up
TRINITY_DN186436_c0_g2	0.006667	1.746667	4.978401	6.96E-19	7.19E-15	up
TRINITY_DN186522_c3_g1	6.22	15.54333	1.102655	6.57E-06	0.003934	up
TRINITY_DN186522_c3_g2	11.49333	24.96333	1.089359	6.51E-08	7.83E-05	up
TRINITY_DN186585_c0_g1	0	3.58	4.387247	4.54E-13	2.00E-09	up
TRINITY_DN186630_c0_g2	0.073333	1.726667	2.625117	4.16E-05	0.016536	up
TRINITY_DN186798_c1_g1	2.976667	8.756667	1.340521	7.32E-05	0.025196	up
TRINITY_DN187048_c0_g1	1.146667	2.866667	1.079193	0.000179	0.046491	up
TRINITY_DN187637_c1_g1	0.056667	3.05	2.96785	3.81E-06	0.002444	up
TRINITY_DN187745_c1_g2	1.16	4.68	1.714028	0.000126	0.036607	up
TRINITY_DN187798_c7_g1	0	17.87333	3.666185	1.19E-08	1.86E-05	up
TRINITY_DN187837_c4_g7	0	2.926667	2.508259	0.000171	0.045055	up
TRINITY_DN187875_c3_g3	0.13	3.723333	3.22193	4.81E-07	0.000452	up
TRINITY_DN188015_c8_g1	0	2.436667	2.875748	1.43E-05	0.007041	up
TRINITY_DN188160_c1_g1	0.09	3.42	3.634402	8.99E-10	1.95E-06	up
TRINITY_DN189264_c8_g1	0.09	1.613333	2.617027	2.98E-05	0.012616	up
TRINITY_DN189636_c1_g1	0.036667	0.656667	3.048784	5.22E-08	6.53E-05	up
TRINITY_DN189636_c1_g2	0.03	0.466667	2.81934	1.66E-06	0.00124	up
TRINITY_DN189883_c7_g3	0	1.84	3.214598	8.63E-07	0.000717	up
TRINITY_DN190374_c6_g1	0.18	5.28	3.313033	9.59E-08	0.00011	up
TRINITY_DN190405_c6_g2	0.093333	1.636667	2.354665	0.00012	0.03552	up
TRINITY_DN190447_c2_g2	0.183333	2.76	2.444069	0.000116	0.034656	up
TRINITY_DN190507_c1_g1	1.29	1.026667	2.848481	6.15E-07	0.000548	up
TRINITY_DN190964_c1_g1	0.02	2.21	3.553731	2.32E-08	3.34E-05	up
TRINITY_DN191398_c5_g1	0.163333	2.963333	2.658557	5.88E-05	0.021354	up
TRINITY_DN191456_c2_g1	8.496667	29.14333	1.165872	7.54E-06	0.004403	up
TRINITY_DN191531_c2_g1	0.153333	3.01	2.379554	0.000156	0.042088	up

TRINITY_DN191932_c0_g1	7.163333	25.23	1.436674	1.27E-09	2.72E-06	up
TRINITY_DN191932_c1_g1	3.043333	9.626667	1.652051	0.000118	0.035065	up
TRINITY_DN192211_c4_g5	0	2.49	3.389446	2.32E-07	0.00024	up
TRINITY_DN192319_c3_g1	1.553333	7.17	1.8035	2.97E-05	0.012611	up
TRINITY_DN192478_c0_g1	12.56	72.04333	1.29865	4.66E-05	0.01792	up
TRINITY_DN192517_c3_g2	11.47333	44.28667	1.617497	2.07E-11	7.34E-08	up
TRINITY_DN192536_c2_g1	8.803333	18.94667	1.028845	0.000101	0.031191	up
TRINITY_DN192923_c3_g2	0.01	0.633333	2.554954	0.00012	0.035453	up
TRINITY_DN192929_c2_g1	1.983333	2.166667	1.843367	7.22E-05	0.024996	up
TRINITY_DN192956_c2_g1	0.023333	2.816667	3.232713	3.00E-07	0.000305	up
TRINITY_DN193024_c0_g2	0.2	5.983333	3.466269	8.73E-10	1.93E-06	up
TRINITY_DN193144_c1_g3	0	0.78	2.509902	0.000176	0.046244	up
TRINITY_DN193587_c2_g1	6.066667	11.22667	1.042335	0.000196	0.049392	up
TRINITY_DN193640_c3_g1	6.596667	17.54	1.308846	5.74E-05	0.021075	up
TRINITY_DN193640_c4_g1	5.733333	26.59	2.017716	6.88E-11	2.24E-07	up
TRINITY_DN193640_c4_g2	7.64	33.50667	2.187367	7.56E-18	6.69E-14	up
TRINITY_DN193640_c4_g3	2.346667	19.13667	2.657125	2.51E-10	6.62E-07	up
TRINITY_DN193650_c3_g2	0.026667	2.95	2.870863	1.64E-05	0.007805	up
TRINITY_DN193706_c3_g4	4.09	21.12333	1.81528	4.88E-10	1.19E-06	up
TRINITY_DN193823_c1_g3	0.14	3.236667	3.027721	5.98E-07	0.000536	up
TRINITY_DN194044_c5_g1	0.203333	8.996667	2.796358	1.01E-05	0.005473	up
TRINITY_DN194145_c3_g1	0.34	1.416667	1.6494	0.000134	0.038412	up
TRINITY_DN194257_c5_g2	3.176667	23.09667	2.427406	6.43E-05	0.022771	up
TRINITY_DN194261_c2_g1	0.413333	94.12	3.507211	1.10E-07	0.000125	up
TRINITY_DN194347_c2_g2	2.716667	9.903333	1.473569	0.000188	0.047998	up
TRINITY_DN194724_c3_g1	3.636667	11.27667	2.070386	5.42E-10	1.29E-06	up
TRINITY_DN195094_c2_g1	1.716667	8.506667	2.382928	1.49E-05	0.007282	up
TRINITY_DN195094_c3_g2	1.966667	3.653333	2.018628	4.18E-05	0.016537	up
TRINITY_DN195481_c11_g1	0.75	2.086667	1.31384	5.09E-07	0.000471	up
TRINITY_DN195581_c3_g4	0.486667	8.303333	2.803574	2.92E-06	0.00199	up
TRINITY_DN195716_c5_g2	0.243333	1.186667	1.869891	2.24E-05	0.010077	up
TRINITY_DN195790_c2_g1	0	72.85333	3.728682	3.99E-09	7.27E-06	up
TRINITY_DN195838_c5_g1	2.526667	7.546667	1.364976	2.34E-05	0.010501	up
TRINITY_DN195920_c1_g1	1.45	9.323333	2.246297	3.89E-05	0.015584	up
TRINITY_DN196256_c9_g1	0.673333	1.646667	1.097942	2.54E-09	5.00E-06	up
TRINITY_DN196502_c8_g1	2.9	22.66	2.692056	2.66E-14	1.50E-10	up
TRINITY_DN196502_c8_g2	1.053333	15.93	2.722652	1.28E-08	1.99E-05	up
TRINITY_DN196506_c30_g3	0.086667	2.486667	2.543105	8.86E-05	0.028644	up
TRINITY_DN20334_c0_g1	0	0.283333	2.488354	0.000197	0.049515	up
TRINITY_DN57967_c0_g1	0	2	2.887491	1.36E-05	0.006773	up

Table S5. Significantly enriched pathways between CG group and TG group.

Num	Pathway Name	Pathway ID	Ratio_in_Study	Ratio_in_pop	P-value
1	Circadian rhythm	map04710	19/197	255/74201	0
2	Circadian rhythm - fly	map04711	12/197	118/74201	0
3	Antigen processing and presentation	map04612	12/197	425/74201	0
4	Herpes simplex infection	map05168	13/197	1167/74201	0
5	Circadian entrainment	map04713	12/197	1147/74201	0.0001
6	Estrogen signaling pathway	map04915	10/197	871/74201	0.0001

7	Th17 cell differentiation	map04659	9/197	719/74201	0.0001
8	Fluid shear stress and atherosclerosis	map05418	11/197	1039/74201	0.0001
9	Tight junction	map04530	13/197	1523/74201	0.0002
10	IL-17 signaling pathway	map04657	7/197	513/74201	0.0005
11	Legionellosis	map05134	6/197	423/74201	0.001
12	Protein processing in endoplasmic reticulum	map04141	10/197	1306/74201	0.0028
13	Hypertrophic cardiomyopathy (HCM)	map05410	7/197	727/74201	0.0035
14	Platelet activation	map04611	9/197	1124/74201	0.0033
15	NOD-like receptor signaling pathway	map04621	9/197	1118/74201	0.0032

Table S6. The primers used in the present study.

**Primers for (quantitative) RT-PCR**	
calm-F: CCATTTCCACCTCAGATTGAC	calm-R: CGGTCTCATGCTAATTTGCC
hsp90-F: GACGCAGTACTCATCGATGG	hsp90-R: GAGCAGTTCTCCAAGAACATC
β-catenin-F: CTAATCCACTGCTTCATCAGC	β-catenin-R: AGGAAAACGAGTGAATGGTG
mek5-F: CGTACCTCTTGAGCCTGAAG	mek5-R: CATTGTGAAACGAAGTGGAC
F-actin-F: GAAGGATATGCTCTTCCACATG	F-actin-R: CTCAATCTTCGAACTGGTAGC
et-1-F: GAGATGGATAACGACAGCATC	et-1-R: CTCGATTAGGTAGCTCTTCACG
th-F: CAGACGTCAGCACACTGGTC	th-R: CTGGATGATCCTGATCCAGATC
dr1-F: TCCTCATCCTGACCACGCTACTG	dr1-F: GCAGTGGAACACATGATGTCAA
dr2-F: CCTATGGCTTGAAGTTTCAGG	dr2-R: GGTCTGCAGCGCTTTCTCTCG
dat-F: TGACATGTGGGAAGGTAGTG	dat-R: TCCTGTGCATCATACAGCGC
mao-F: CATGTCATGGAACTTATCCGAG	mao-R: CCATCATACAGCTCAGCGTTAG
ef1-F: GAAGAACGTGTCTGTCAAGG	ef1-R: GTTCAGGATGATGACCTGA

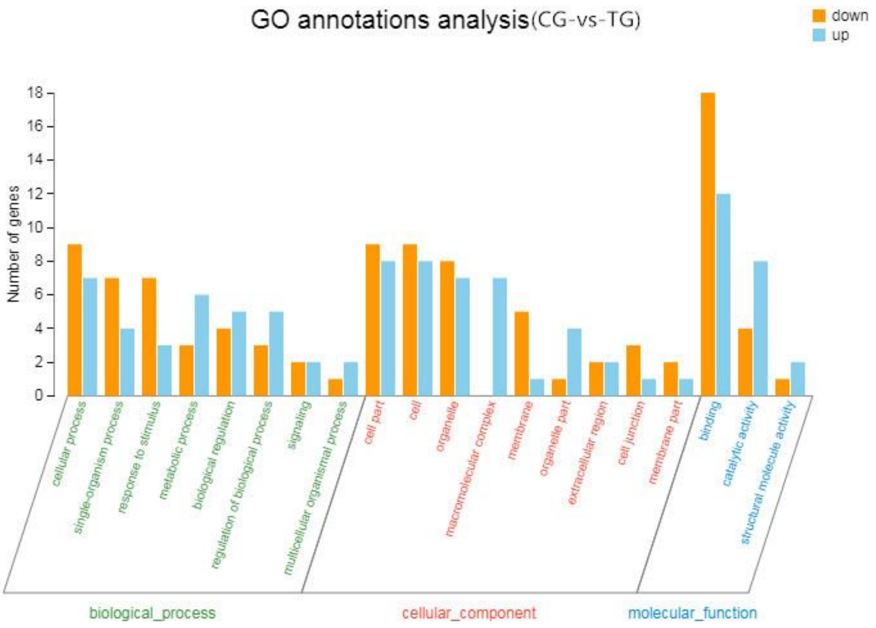

Figure S1. Enriched GO terms for the DEGs between the control group (CG) and the BPA exposure group (TG).

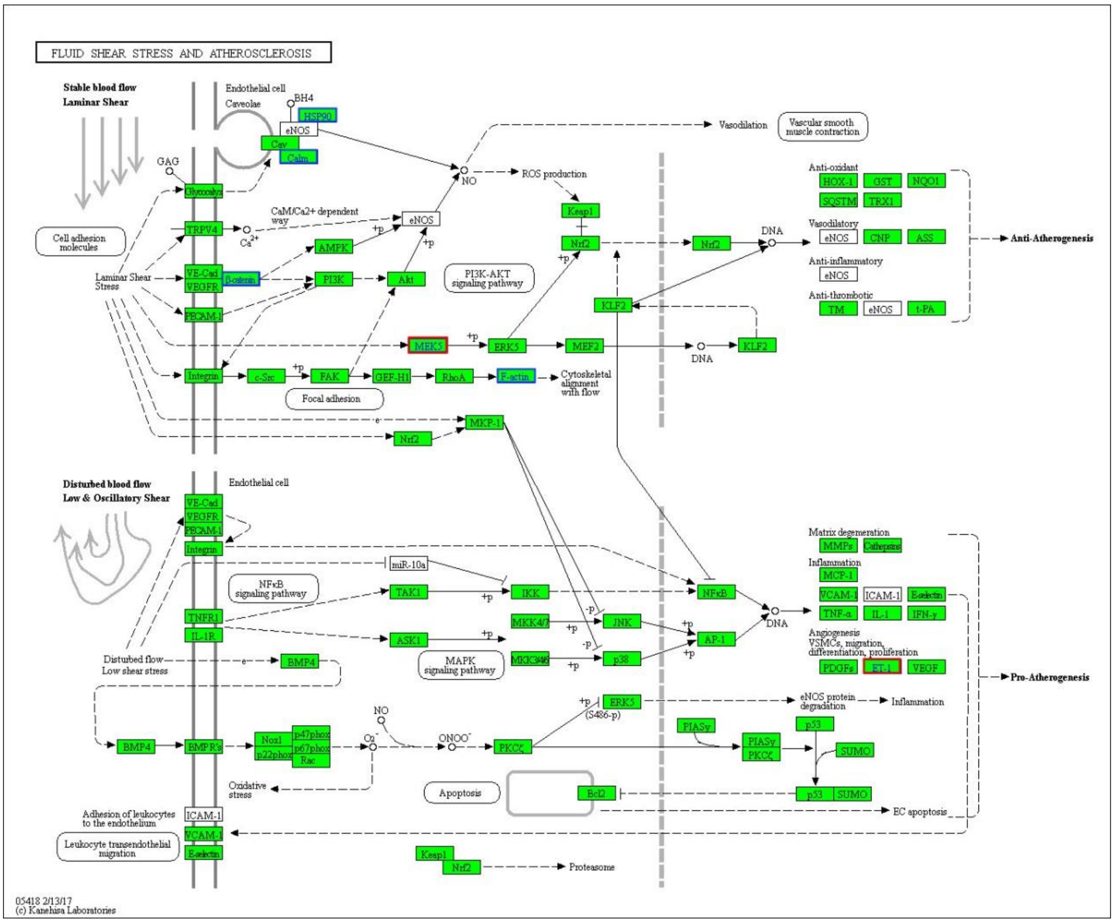

Figure S2. Enriched DEGs in the fluid shear stress and atherosclerosis pathway. The DEGs included in the control group (CG) vs BPA exposure group (TG) comparison. Blue indicates a decrease in expression in the BPA exposure group compared to the control group; red indicates an increase in expression in the BPA exposure group compared to the control group.

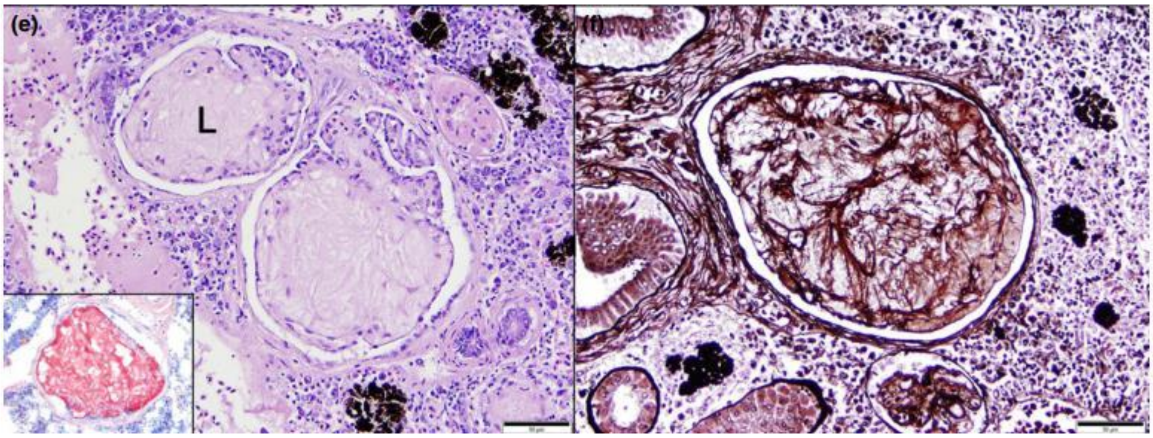

Figure S3. Images of the H&E staining of atherosclerotic pathology in *Paralichthys albigutta* from Dill et al., 2017. (**e**) Kidney, H&E stain. Two glomeruli are effaced by segmental to global solidification of capillary tufts by vacuolated material, identified as lipid with Oil Red O (inset), traversed by fine eosinophilic fibres (L). (**f**) Kidney, Jone's methenamine silver stain. Argyrophilic fibrillar (black) remnants of glomerular basement membrane and mesangial matrix disrupted by lipid deposition.

Dill1, J.; Brown, C.; Heym, K.; Camus, A. Lipoid liver disease, atherosclerosis and glomerular lipidosis in a Gulf flounder Paralichthys albigutta (Jordan & Gilbert 1882): a case report. *J. Fish. Dis*. **2017**, *40*, 273–278.
